# Phosphate Transporter *BnaPT37* Regulates Phosphate Homeostasis in *Brassica napus* by Changing Its Translocation and Distribution *In Vivo*

**DOI:** 10.3390/plants12193362

**Published:** 2023-09-22

**Authors:** Yu Li, Xue Wang, Hao Zhang, Xiangsheng Ye, Lei Shi, Fangsen Xu, Guangda Ding

**Affiliations:** College of Resources and Environment, Microelement Research Center, Key Laboratory of Arable Land Conservation (Middle and Lower Reaches of Yangtze River), Ministry of Agriculture and Rural Affairs, Huazhong Agricultural University, Wuhan 430070, China; liyu1@webmail.hzau.edu.cn (Y.L.); wangxueer@webmail.hzau.edu.cn (X.W.); hao.zhang@webmail.hzau.edu.cn (H.Z.); xiangshengye@mail.hzau.edu.cn (X.Y.); leish@mail.hzau.edu.cn (L.S.); fangsenxu@mail.hzau.edu.cn (F.X.)

**Keywords:** *Brassica napus*, phosphate transporter, *BnaPT37*, phosphate translocation, phosphate distribution

## Abstract

Inorganic phosphate (Pi) is actively taken up by Pi transporters (PTs) from the soil and transported into the plant. Here, we functionally characterized the *Brassica napus* gene *BnaPT37,* which belongs to the PHT1 family. BnaPT37 is a plasma membrane-localized protein containing 534 amino acids. Expression of *BnaPT37* increased significantly under Pi deficiency in various tissues, especially in fully expanded leaves. Expression of the β-glucuronidase reporter gene driven by the *BnaPT37* promoter showed that *BnaPT37* is expressed in the root, stem, calyx, and leaf under Pi deficiency. *BnaPT37* can complement a yeast mutant strain defective in five Pi transporters and can restore the growth of the *Arabidopsis atpt1/2* double mutant under Pi deprivation. Overexpression of *BnaPT37* in rapeseed significantly increased Pi translocation from root to shoot. Moreover, the movement of Pi from fully expanded leaves to new leaves and roots was enhanced in the transgenic lines compared to the wild type. However, the overexpression of *BnaPT37* inhibited the flowering time, plant height, and Pi accumulation in seeds. In conclusion, BnaPT37 functions as a plasma membrane-localized Pi transporter and might be involved in Pi translocation from root to shoot and Pi distribution from source to sink in *B. napus*.

## 1. Introduction

Phosphorus (P) is one of the essential macronutrients for plant growth and development. P is an important component of phosphate (Pi), nucleoprotein, phospholipid, and phytate and is involved in various metabolic activities in plants, such as photosynthesis, carbohydrate metabolism, fat metabolism, and so on [1]. Moreover, P can improve the drought resistance and disease resistance of plants [2,3,4]. In soil, there is both organic and inorganic P; plants, however, mainly absorb inorganic P [5]. Although the total P content is very high in soil, about 80% of P exists in the organic form, and Pi ions are easily fixed by the soil, which makes it difficult for plants to use [6]. Therefore, the phytoavailability of P in soil is often very low. P fertilizer can provide sufficient nutrients for crops, but the input of a large amount of P fertilizer may result in environmental pollution, such as eutrophication in water systems, and damage the environmental ecosystem [7]. In addition, the massive use of P fertilizer accelerates the depletion of Pi rock, a non-renewable resource [8,9].

When the soil is short of P, that is, the content of available P in the soil solution is less than 10 μM [10], the plant may produce a series of morphological adaptive changes to cope, such as changing root architecture, increasing root exudation, and inducing the expression of P starvation response genes [9,11]. During P deficiency, plants can induce the expression of Pi transporters (PHTs) to uptake P from the rhizosphere soil. The high-affinity P transporters can absorb P at very low external P concentrations [12,13]. The PHT1 family belongs to the ninth subfamily of the major facilitator superfamily (MFS). It is strongly induced by low P stress, involved in P absorption by plant roots and transport in plants, and is extremely important for plant growth [14,15,16]. The PHT1 family genes contain 12 transmembrane domains (TMs) and generally have a hydrophilic ring between the sixth and seventh transmembrane domains [17,18]. The PHT1 family mainly transports Pi from soil to root cells through Pi/H^+^ co-transport, and there are significant differences in the characteristics of Pi transport among its members [19,20,21,22,23].

PHT1 family genes play important roles in Pi uptake and transport in plants. The *Arabidopsis* PHT1 family has nine members, and eight of them are mainly expressed in the root [19]. Under high and low P conditions, the Pi uptake efficiency of the *pht1;1* and *pht1;4* mutants were significantly lower than that of the wild type, and the dual mutants exhibited additive effects, indicating that *AtPHT1;1* and *AtPHT1;4* play a key role in Pi uptake [14,24,25]. *AtPHT1;5* showed a higher transcript level in leaves than that in roots under P-deficient conditions, mediating P translocation between source and sink organs, and its mutation affected P transport to the shoot [26], while *AtPHT1;8* and *AtPHT1;9* were involved in Pi translocation from root to shoot [15]. In addition, *AtPHT1;8* and *AtPHT1;9* might participate in Pi translocation together with *AtPHT1;3* and *AtPHT1;4* [19]. In rice (*Oryza sativa*), there are 13 members of the PHT1 family [13]. *OsPHT1;1*, expressed constitutively in the root and the shoot, was involved in Pi uptake and transport under P-sufficient conditions [27]. OsPHT1;2 is a low-affinity P transporter expressed in the main root of the central column of lateral root, mediating Pi transfer into root vasculature [20]. *OsPHT1;3* was strongly induced by an extremely low P stress condition, and the P absorption efficiency of the *ospht1;3* mutant significantly decreased compared to the wild type [28]. The Pi uptake ability of *OsPHT1;8* RNAi lines was significantly reduced compared to the wild type, indicating its role in mediating Pi uptake in rice [29,30]. Overexpression of *OsPHT1;9* and *OsPHT1;10* significantly increased the Pi uptake rate of rice, while the inorganic P concentration of the double mutant significantly decreased [16]. *OsPHT1;7* is highly expressed in anthers and plays an important role in P transport, redistribution, and anther development [31]. The mutation of *OsPHT1;7* significantly reduces P redistribution into new leaves, leaving more P stuck in old leaves, leading to a significant decrease in yield and P concentration in the anthers [31]. *OsPHT1;4* is strongly expressed in roots and embryos and plays an important role in embryonic development [32]. Both *OsPHT1;11* and *OsPHT1;13* are specifically induced by mycorrhizal fungi, and are involved in Pi uptake by the arbuscular mycorrhizal symbiotic pathway [33,34]. Similar to *Arabidopsis*, there were collaborations between Pi transport proteins in rice; for example, the function of *OsPHT1;2* was probably enhanced via other OsPHT family members [35]. A total of 13 PHT1 family members have been identified in maize, and *ZmPHT1;7* plays a central role in Pi uptake as well as in P redistribution from old to young leaves [36].

*Brassica napus* is an important oil crop worldwide, but it is sensitive to P stress and needs massive amounts of P for high seed yield and oil content [37]. In addition, *B. napus* has suffered from a constant P deficiency worldwide [38]. Therefore, it is crucial to breed *B. napus* cultivars with increased P-use efficiency and improved seed production. The PHT1 family genes play an important role in Pi uptake and translocation in plants [12,13]. In our previous research, we found that there are 49 members of the PHT1 family in *B. napus* with different expression patterns both in the root and shoot, indicating that the rapeseed PHT1 family genes may play different roles in P homeostasis in *B. napus* [39]. The reported *BnPHT1;4* gene localizes in the plasma membrane, is induced by P deficiency in the root, and is expressed in the early cotyledon [40]. Overexpression of *BnPHT1;4* significantly reduces P accumulation in cotyledons while increasing inorganic P content and promoting seed germination, indicating that *BnPHT1;4* is involved in plant P uptake and early seed germination [41]. In this study, *BnaPT37*, a member of the PHT1 family that was induced by P deficiency in root and shoot, was isolated and characterized in *B. napus*. To investigate the function of *BnaPT37* in P homeostasis, we developed transgenic lines with overexpression of *BnaPT37* in *B. napus*. Our results revealed that *BnaPT37* is involved in Pi uptake and the distribution of Pi to new leaves.

## 2. Results

### 2.1. Sequence Alignment and Phylogenetic Analysis of BnaPT37 Gene

**Figure 1:**
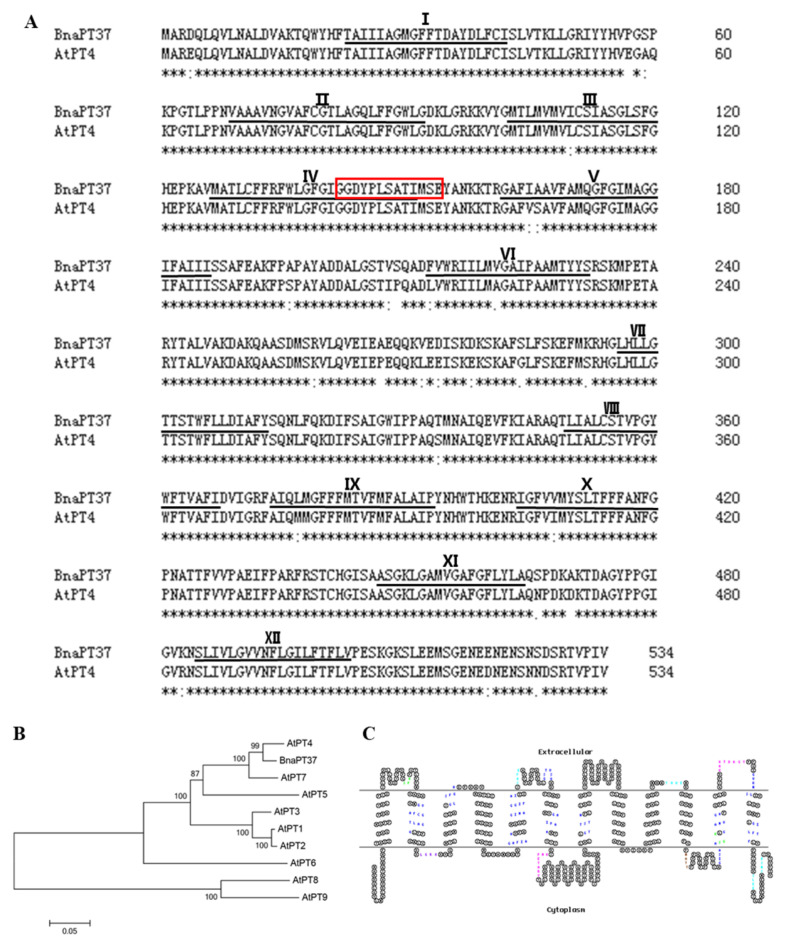
Characterization of *BnaPT37* and phylogenetic relationship with other phosphate (Pi) transporters in *Arabidopsis*. (**A**) Alignment of amino acid sequences between BnaPT37 and AtPT4. Amino acid sequences are aligned by Clustal Omega. The predicted 12 transmembrane domains of *BnaPT37* were underlined and numbered as I–XII. The prediction of transmembrane domains was carried out using HMMTOP. The red box is the conserved PHT1 signature (GGDYPLSATIMSE). (**B**) The phylogenetic relationship of *BnaPT37* with other Pi transporters in *Arabidopsis*. The minimum evolution tree was constructed using MEGA7 with 1000 bootstrap replicates. (**C**) The transmembrane model was drawn using TOPO2 website The significant domains are predicted by Expasy. Enlarged symbols indicate sites of significant structure-function importance: brown, N-glycosylation; green, protein kinase C phosphorylation; cyan, casein kinase II phosphorylation; magenta, tyrosine kinase phosphorylation; purple, amidation; and blue, N-myristoylation. * indicates conservative amino acid sequence.

There are 49 members of the putative phosphate transporter PHT1 family in *B. napus* named *BnaPT1*–*BnaPT49* [39]. Sequence analysis showed that the coding sequence (CDS) of the *BnaPT37* gene consists of 1605 bp. The protein sequence of BnaPT37 contains 534 amino acids, which share about 95% of their identity with AtPT4 in *Arabidopsis* (Figure 1A,B). The protein structure of BnaPT37 is similar to that of AtPT4, both of which contain 12 transmembrane domains and a conserved PHT1 signature (GGDYPLSATIMSE) (Figure 1A,C and Figure S1).

### 2.2. The Expression Pattern of BnaPT37 in Response to Different P Supplies

**Figure 2:**
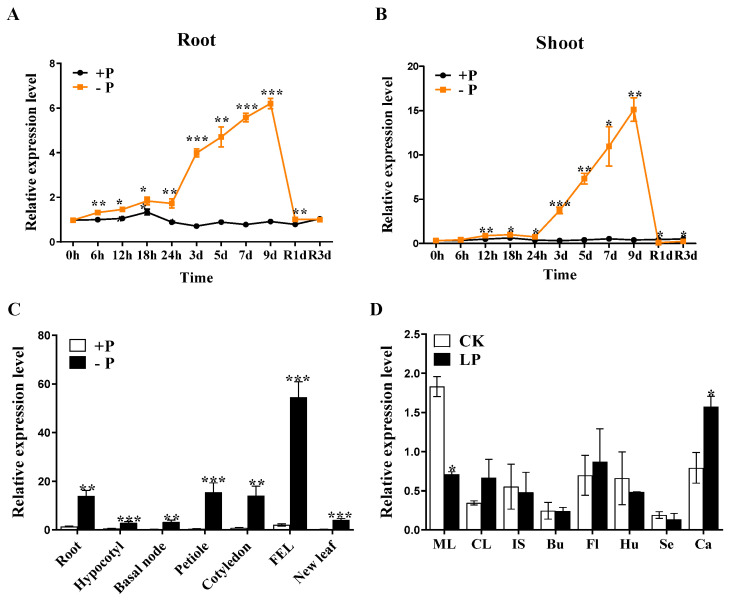
Expression pattern of *BnaPT37* in response to phosphate (Pi) starvation in *Brassica napus*. (**A**,**B**) Gene expression patterns in the root (**A**) and shoot (**B**) in a time-course treatment. Twelve-day-old seedlings were treated with +P (1 mM Pi) or −P (0 mM Pi) for 9 days, then resupplied with 1 mM Pi for 3 days. The root and shoot of the plants were sampled at 0 h, 6 h, 12 h, 24 h, 3 days, 5 days, 7 days and 9 days after the treatment, and 1 day (R1d) and 3 days (R3d) after the resupply of Pi. (**C**) Gene expression in different organs at seedling stage. Two-week-old seedlings were transferred into +P (1 mM Pi) or −P (0 mM Pi) for 7 days. The root, hypocotyl, basal node, petiole, cotyledon, fully expanded leaf (FEL), and new leaf were sampled. (**D**) Gene expression in different organs at the reproductive stage. The mature leaf (ML), cauline leaves (CL), inflorescence stems (IS), bud (Bu), flower (Fl), Husk (Hu), Seeds (Se), and carpopodium (Ca) were sampled separately at the ripen stage after CK (90 kg ha^−1^ P_2_O_5_) and LP (15 kg ha^−1^ P_2_O_5_) treatments in a field. Gene expression levels were determined by RT-qPCR. *BnaEF1-a* (Accession number DQ312264) was used as an internal control. Values represent means ± SD of biological replicates (*n* = 3). Data significantly different from those in the corresponding controls are indicated as * *p* < 0.05, ** *p* < 0.01, and *** *p* < 0.001 by Student’s *t* test.

Most of the reported PHT1 family members are regulated by the external P environment, mainly by P deficiency [13]. A time-course treatment was performed to investigate the responses of *BnaPT37* expression to a Pi shortage signal. In roots, *BnaPT37* was significantly induced by Pi starvation after 6 h of treatment (Figure 2A). The induction rate grew steadily throughout the course of the treatment, peaking at Day 9, and a 1-day resupply of Pi reduced *BnaPT37* expression to levels equivalent to those observed before P stress treatment (Figure 2A). In shoots, the induction of *BnaPT37* expression was observed after 12 h of P deprivation treatment. It reached its maximum after 9 days of P stress treatment and decreased sharply to those at the beginning of P stress treatment after a 1-day resupply of Pi (Figure 2B).

In order to further clarify the expression pattern of the *BnaPT37* gene in different tissues, we divided *B. napus* seedlings into seven tissues, including the root, hypocotyl, basal node, petiole, cotyledon, fully expanded leaf, and new leaf, to determine the expression level of the *BnaPT37* transcript. Consistent with the expression pattern at different time points, *BnaPT37* was significantly up-regulated by P deficiency in all tissues, but the highest expression was observed in the fully expanded leaf under P deficiency, followed by the petiole, root, and cotyledon (Figure 2C). The expression pattern of the *BnaPT37* gene in the maturing stage was also analyzed. The result showed that *BnaPT37* was highly induced by low-P treatment in carpopodium but inhibited in fully expanded leaves. No significant difference in *BnaPT37* expression was observed in other tissues under both P treatments (Figure 2D).

### 2.3. BnaPT37 Is P-Starvation-Induced and Plasma Membrane-Localized Protein

**Figure 3:**
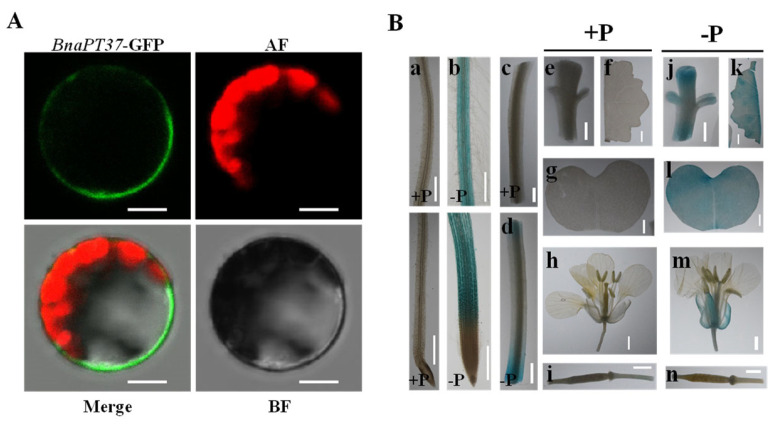
Subcellular and tissue-specific localization of BnaPT37 protein. (**A**) Subcellular localization of BnaPT37. Constructs for C-terminal fusion of BnaPT37 with green fluorescent protein (GFP) were transformed into *Arabidopsis* protoplasts. The green signals indicate GFP, and the red signals indicate chloroplast auto-fluorescence (AF). Scale bars = 8 μm. BF, bright field; (**B**) *pBnaPT37:GUS* transgenic rapeseed plants were cultured at +P (1 mM Pi) and −P (0 μM Pi) for seven days, and stained at root (**a**,**b**), stem (**c**,**d**), node (**e**,**j**), leaf (**f**,**k**), cotyledon (**g**,**l**), flower (**h**,**m**) and silique (**i**,**n**) under +P (**a**,**c**,**e**–**i**) and −P (**b**,**d**,**j**–**n**). Shoot bar = 2 mm. Root bar = 0.5 mm.

We transiently inserted BnaPT37 fused with GFP into *Arabidopsis* leaf protoplasts under the control of the 35S promoter to examine the subcellular location. The results showed that the GFP signal of BnaPT37-GFP was identified as a fine ring at the cell periphery (Figure 3A), indicating that BnaPT37 is a plasma membrane-localized protein.

To investigate the tissue specificity and Pi responsiveness of *BnaPT37*, we generated transgenic *B. napus* lines carrying a 2 kb promoter region of *BnaPT37* fused with the β-glucuronidase (GUS) reporter gene. The results showed that GUS driven by the *BnaPT37* promoter was highly expressed in various cells of the root, stem, and leaf under P-deficient conditions (Figure 3B). In addition, the expression of *BnaPT37* was also detected in the calyx under P-deficient conditions at the flowering stage but not in the young pod (Figure 3B).

### 2.4. BnaPT37 Mediates Pi Transport in Yeast and in Arabidopsis

**Figure 4:**
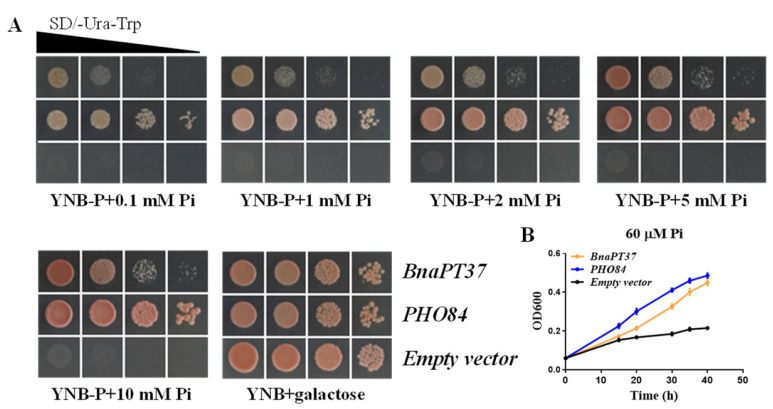
Evaluation of the phosphate (Pi) transport activity of BnaPT37 in a yeast mutant. (**A**) Complementation of yeast mutant EY917 (Δ*pho84*Δ*pho87*Δ*pho89*Δ*pho90*Δ*pho91*) defective in Pi uptake by BnaPT37. Yeast cells harboring either the *PHO84* cNDA construct (positive control) or an empty expression vector (negative control) and the *BnaPT37* cDNA construct were grown in a synthetic dropout (−Ura/−Trp) liquid medium containing a 2% (*w*/*v*) galactose to OD600 = 1. Equal volumes of 10-fold serial gradient dilutions were applied to YNB without the Pi (YNB-P; pH 5.5) medium with different Pi concentrations and a 2% (*w*/*v*) glucose, and incubated at 28 °C for 4 d. (**B**) Growth curve of EY917 transformed with *BnaPT37*, *PHO84*, and an empty expression vector under a 60 μM Pi medium. The yeast solution was washed with sterile water and adjusted to 1 under OD600. Then, 40 μL aliquots were added to 2 mL of a synthetic dropout (−Ura/−Trp) liquid medium containing a 60 μM Pi and a 2% (*w*/*v*) glucose. The culture was incubated at 28 °C, and the OD600 was measured by sampling at different time points.

To acquire biochemical support for the function of *BnaPT37*, we conducted complementation tests using the yeast mutant strain EY917, which is defective in five Pi transporters (PHO84, PHO89, PHO87, PHO90, and PHO91) [42,43]. At the same time, it can activate the Gal-inducible promoter to express the yeast Pi transporter gene *PHO84* in the presence of galactose, so that the yeast mutant EY917 can absorb Pi in the medium and resume growth (Figure 4A). In addition, *PHO84*, a reported yeast Pi transporter gene, was used as the positive control. On the defective growth medium with glucose as the carbon source and different Pi concentrations, the transformed cells with either *BnaPT37* or *PHO84* grew very well on the medium with different Pi concentrations, but the yeast transformed with an empty vector was completely inhibited (Figure 4A). Compared to the transformed yeast cells with *BnaPT37*, the cells with *PHO84* could grow better. Then, the growth curves of yeast at different time points were further verified. At 60 μM Pi, the growth state of yeast expressing *BnaPT37* is slightly lower than that of yeast expressing *PHO84* but still much higher than that of yeast expressing an empty vector (Figure 4B). These results indicate that *BnaPT37* can partially restore the growth of yeast mutant EY917.

To further confirm that *BnaPT37* can transport Pi, *BnaPT37* was transformed into the *Arabidopsis atpt1/2* double mutant that lacks the main Pi transporters *AtPHT1;1* and *AtPHT1;4*. Three independent complementary lines were selected for phenotypic analysis (Figure S2A). Under 15 μM P treatment, the root and shoot fresh weights of the complementary materials were significantly higher than those of the *atpt1/2* double mutant and wild type, while the shoot fresh weights of the complementary materials were slightly higher than those of the *atpt1/2* double mutant under 625 μM P conditions (Figure S2B–D). The inorganic P concentrations in the shoot and root of the complementary materials were significantly higher than those of the *atpt1/2* double mutant, even higher than those of the wild-type plants under both high- and low-P treatments (Figure S2E,F). These results indicate that *BnaPT37* has Pi uptake activity and can recover the P-deficient phenotype of the *Arabidopsis atpt1/2* double mutant. These results suggest that *BnaPT37* is a phosphate transporter.

### 2.5. Overexpression of BnaPT37 Retards Rapeseed Growth at Seedling Stage

**Figure 5:**
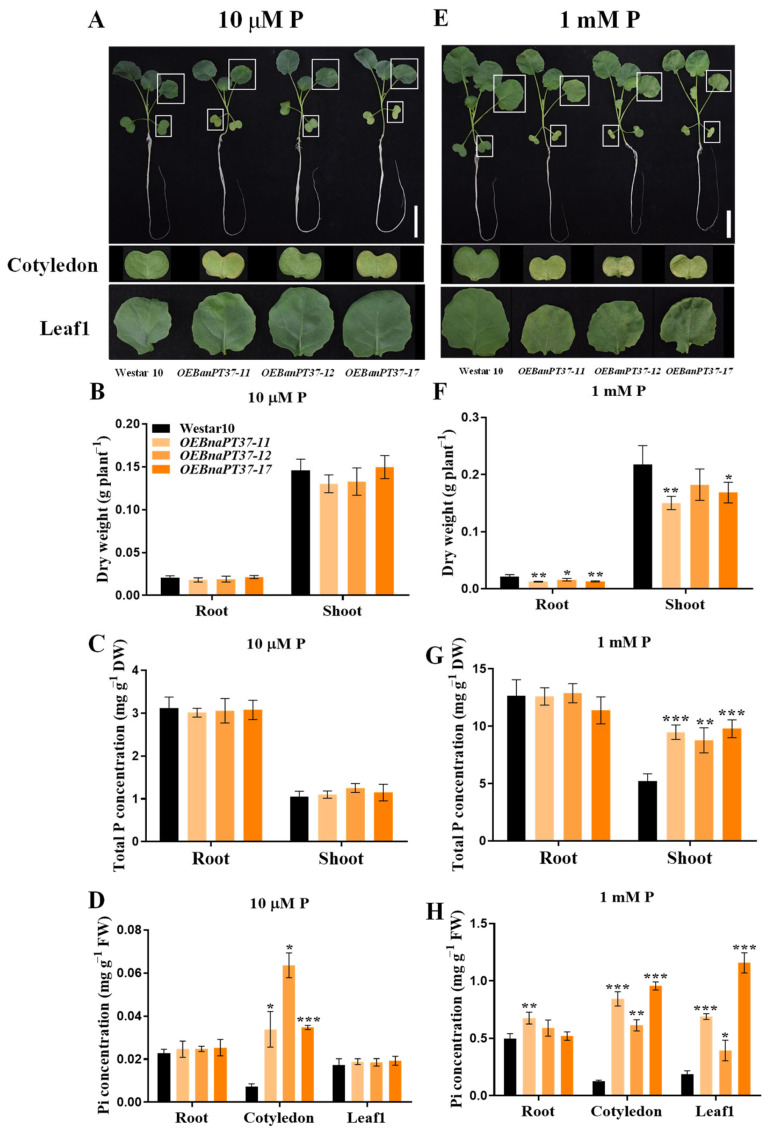
Overexpression of *BnaPT37* affects rapeseed growth and phosphorus (P) concentration under two contrasting P conditions. The 5−day−old seedlings of wild-type and *BnaPT37* overexpression plants were transferred to 10 μM and 1 mM P conditions for 14 d. Then, the phenotypes (**A**,**E**), dry weight (**B**,**F**), total P concentration (**C**,**G**), and Pi concentration (**D**,**H**) were investigated under 10 μM (**A**−**D**) and 1 mM (**E**−**H**) P conditions. Error bars indicate standard deviation (*n* = 4). Data significantly different from those in the corresponding controls are indicated as * *p* < 0.05, ** *p* < 0.01, and *** *p* < 0.001 by Student’s *t* test.

To determine the functions of *BnaPT37* in plant growth and Pi transportation, three independent *BnaPT37* homozygous overexpression lines were created. Reverse-transcription PCR (RT-PCR) was used to confirm the expression levels of *BnaPT37* in the three overexpression lines (Figure S3). After treatment with low P conditions, the overexpression lines showed more severe chlorosis and necrosis on the cotyledon of rapeseed compared to the wild-type plants. The same phenotype was observed on both the cotyledon and leaf1, which is the first true leaf of rapeseed under high P supplies (Figure 5A,E). We then detected the SPAD value and the contents of chlorophyll a, chlorophyll b, and carotenoid in the cotyledon and leaf1 of both transgenic and wild-type plants under high and low P stress environments. The results showed that they were lower in the cotyledon of the *BnaPT37* overexpression lines than in the wild type under two P levels (Figure S4). In addition, the root and shoot dry weight of the transgenic plants was much lower than that of the wild-type plants under high-P conditions, but no significant differences were detected under P-deficient conditions (Figure 5B,F).

### 2.6. Pi Translocation and Distribution Changes in the BnaPT37 Transgenic Plants

**Figure 6:**
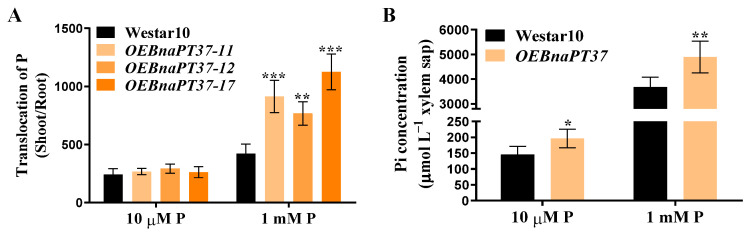
Overexpression of *BnaPT37* increased the root-to-shoot translocation of phosphorus (P) in *Brassica napus*. The 5−day−old seedlings were transplanted into 1 mM and 10 μM P for 14 d. (**A**) The root−to−shoot translocation of P (*n* = 4); (**B**) Pi concentration in the xylem sap of Westar10 and *BnaPT37* overexpression lines under two P treatments (*n* = 5). Error bars indicate the standard deviation. Data significantly different from those in the corresponding controls are indicated as * *p* < 0.05, ** *p* < 0.01, and *** *p* < 0.001 by Student’s *t* test.

Higher total P concentrations were observed only in the shoots of the three overexpression lines under high-P conditions compared to wild-type plants (Figure 5C,G). Furthermore, the inorganic P concentrations in the cotyledon and leaf1 of the overexpression lines were significantly higher than those in wild-type plants under normal-P conditions, whereas higher inorganic P concentrations were detected only in the cotyledons of the overexpression lines than in the wild-type plants under low-P treatments (Figure 5D,H). However, overexpression of *BnaPT37* had little effect on the P accumulation in the whole plant, and only the root P accumulations of the overexpression lines were slightly reduced under high-P conditions (Figure S5). We then further calculated the root-to-shoot translocation of P and the P concentration in the xylem sap. Under high-P treatment, the Pi translocation from root to shoot of the overexpression lines increased significantly compared to the wild-type plants, and similar results were observed for the P concentrations in the xylem sap under both P treatments (Figure 6). These results indicate that overexpression of *BnaPT37* may enhance rapeseed Pi translocation from root to shoot.

**Figure 7:**
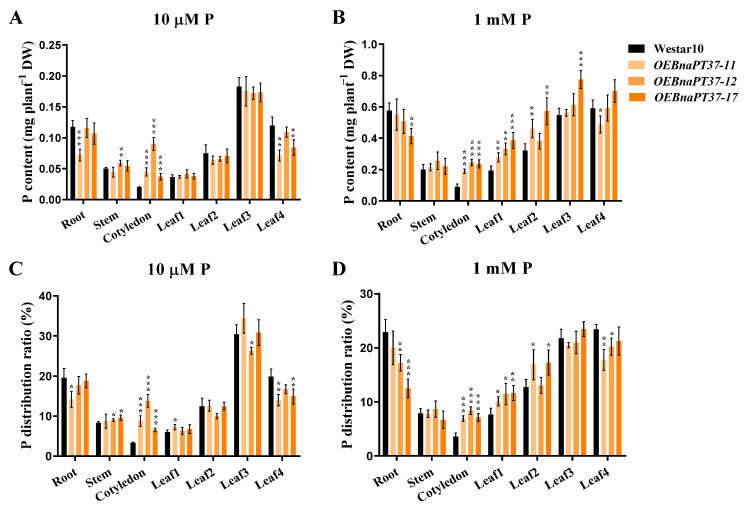
Overexpression of *BnaPT37* affects the allocation of phosphorus (P) to new leaves in *Brassica napus*. (**A**,**B**), The total P content in different tissues of Westar10 and *BnaPT37* overexpression plants under low (**A**) − and high (**B**) −P conditions. The 5−day−old seedlings were transferred to 10 μM and 1 mM P until the fourth leaf appeared (about 16 d). The root, stem, cotyledon, and four leaves (leaf1 to leaf4) were sampled separately for the determination of P content; (**C**,**D**), P distribution of of Westar10 and *BnaPT37* overexpression plants under low (**C**) − and high (**D**) −P conditions. Leaf1 indicates the oldest leaf, and leaf4 indicates the youngest leaf. Error bars indicate the standard deviation (*n* = 4). Data significantly different from those in the corresponding controls are indicated as * *p* < 0.05, ** *p* < 0.01, and *** *p* < 0.001 by Student’s *t* test.

The Pi concentrations in the cotyledon and leaf1 of the overexpression lines increased significantly compared to those in the wild type (Figure 5), suggesting that overexpression of *BnaPT37* may affect P distribution in plants. Thus, we analyzed the P contents and distributions in different tissues of the overexpression lines and the wild type. The P content increased in the cotyledon of the overexpression lines compared to the wild-type plants but decreased in leaf 4, the youngest leaf, under low-P conditions (Figure 7A). Under high-P conditions, higher P contents were observed in the cotyledon, leaf1 and leaf2 of the overexpression lines compared to those of the wild type (Figure 7B). Then, we calculated the P distribution ratio among different tissues of the plants. It showed that P distribution in the cotyledon of the overexpression lines was significantly higher than in the wild type under low-P treatment but lower in the newest leaf 4 (Figure 7C). Under high P levels, the distribution of P was high in cotyledon leaf1 and leaf2 but lower in leaf4 of the *BnaPT37* overexpression lines compared to that of the wild type (Figure 7D). These results suggest that *BnaPT37* may be involved in P allocation in *B. napus*.

**Figure 8:**
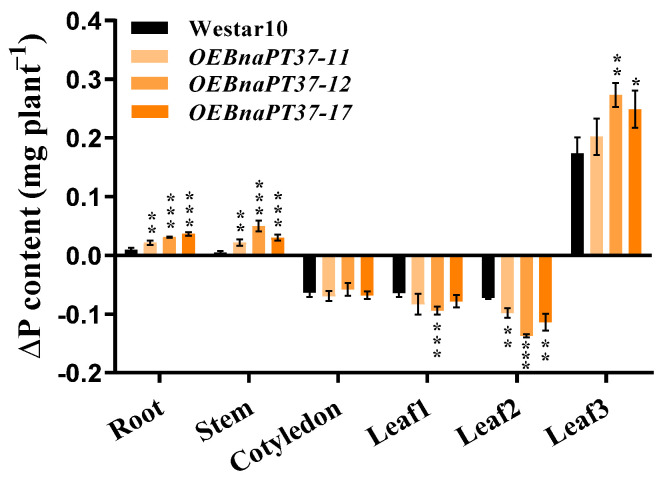
Redistribution of phosphorus (P) in the *BnaPT37* overexpression lines and the wild−type (Westar10) plants. The 5−day−old Westar 10 and *BnaPT37* overexpression seedlings were transferred to a nutrient solution with 1 mM P until leaf2 appeared. Then, all the plants were transferred to a nutrient solution without P for 6 d. Different tissues, including the root, stem, cotyledon, leaf1, leaf2, and leaf 3, were harvested separately before and after P starvation treatment. The difference in P content (∆P) of each part was calculated. Error bars indicate the standard deviation (*n* = 4). Data significantly different from those in the corresponding controls are indicated as * *p* < 0.05, ** *p* < 0.01, and *** *p* < 0.001 by the Student’s *t* test.

To further check whether *BnaPT37* functions in P redistribution, we cultivated the transgenic and wild-type plants under high-P environments for 15 d and then transferred them to P-free treatments for 6 d. The P concentrations and contents in different tissues were analyzed before and after P-free treatments. We found that ΔP contents were higher in the newest leaf 3, root, and stem but lower in fully expanded leaf2 and leaf1 of the *BnaPT37* overexpression lines compared to the wild type (Figure 8), indicating that overexpression of *BnaPT37* accelerated P reallocation from source to sink organs in *B. napus*.

### 2.7. Overexpression of BnaPT37 Affects Plant Height and P Distribution at the Flowering Stage

**Figure 9:**
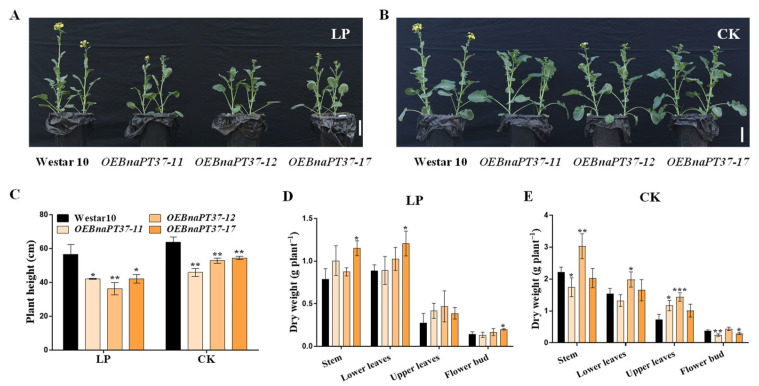
Phenotyping, plant height, and dry weight of Westar10 and *BnaPT37* overexpression lines at the flowering stage. (**A,B**) The growth performance. (**C**) Plant height. (**D**) Dry weight under low P (LP) supply. (**E**) Dry weight under normal P (CK) supply. The Westar10 and *BnaPT37* overexpression plants were grown under CK (P_2_O_5_, 150 mg kg^−1^) and LP (P_2_O_5_, 20 mg kg^−1^) conditions until flowering in a pot trial. The stem, lower leaves, upper leaves, and flower bud were sampled separately. Scale bars = 10 cm. Each treatment contained four biological replicates. Error bars indicate the standard deviation. Data significantly different from those in the corresponding controls are indicated as * *p* < 0.05, ** *p* < 0.01, and *** *p* < 0.001 by Student’s *t* test.

*BnaPT37* is expressed highly in rapeseed shoots (Figure 2), and overexpression of *BnaPT37* affects P allocation in rapeseed seedlings (Figure 6, Figure 7 and Figure 8). Thus, we speculated that *BnaPT37* may also affect rapeseed growth at the reproductive stage. Then, we planted the transgenic lines and wild type using a pod culture system in real soil. The flowering time of the overexpression lines was delayed compared to the wild type under both sufficient and deficient P supplies (Figure 9A,B), while the plant height of the overexpression lines was lower than that of the wild-type plants (Figure 9C). However, the dry weight of the stem, lower leaves, and upper leaves of the overexpression lines was slightly higher than that of the wild-type plants (Figure 9D,E). In addition, overexpression of *BnaPT37* had little effect on P concentration in various rapeseed tissues (Figure S6A,D). Nevertheless, the P distribution was significantly inhibited in flower buds but enhanced in the upper leaves of *B. napus* by overexpression of *BnaPT37* under sufficient P supplies (Figure S6F).

### 2.8. BnaPT37 Is Involved in the Translocation of P into Grains at the Ripening Stage

**Figure 10:**
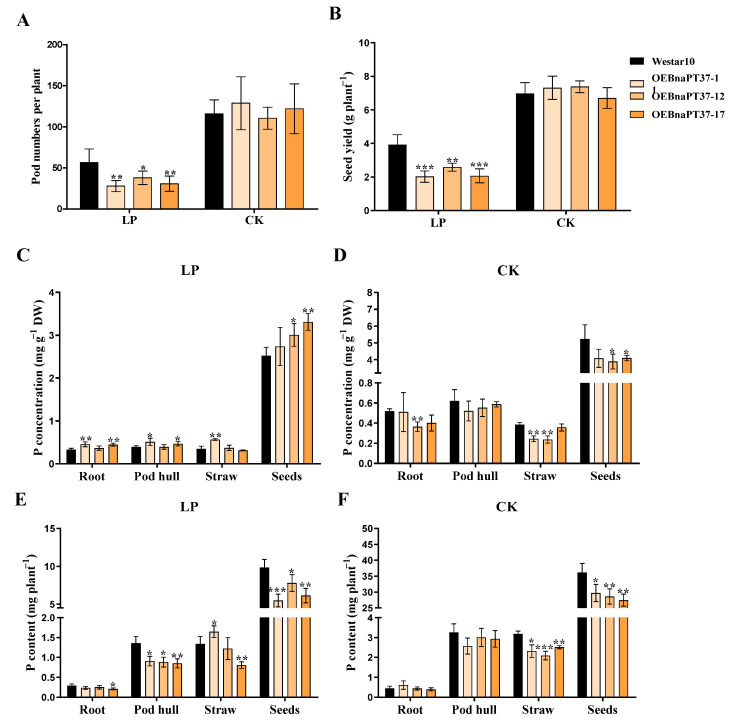
Overexpression of *BnaPT37* reduced phosphorus (P) transport to the seeds under both normal (CK) and low (LP) P supplies at the ripening stage. (**A**) Pod numbers per plant; (**B**) seed yield per plant; (**C**) P concentration under low−P conditions; (**D**) P concentration under sufficient−P conditions; (**E**) P content under low-P conditions; (**F**) P content under sufficient−P conditions. The Westar10 and *BnaPT37* overexpression plants were grown under CK (P_2_O_5_, 150 mg kg^−1^) and LP (P_2_O_5_, 20 mg kg^−1^) conditions until the seed ripened in a pot trial. Different tissues of Westar10 and *BnaPT37* transgenic plants, including root pod hull, straw, and seeds, were harvested separately for the determination of P concentrations and contents. Each treatment contains four biological replicates. Error bars indicate the standard deviation. Data significantly different from those in the corresponding controls are indicated as * *p* < 0.05, ** *p* < 0.01, and *** *p* < 0.001 by Student’s *t* test.

Seed yield and agronomic performance were investigated at the ripening stage. The plant height, first branch height, branch number, seed number per pod, and thousand-seed weight were not affected by the overexpression of *BnaPT37* under both P levels (Figure S7A–G). However, pod numbers in the main inflorescence and lateral branches were significantly reduced in the transgenic lines compared to the wild type under low-P treatment, as was the straw weight (Figure S7H–K). In addition, overexpression of *BnaPT37* significantly reduced both seed yield and pod number per plant under low-P treatment, while no difference was observed under normal-P treatment (Figure 10A,B). We further determined the P concentrations and contents in different organs of the *BnaPT37* overexpression lines and wild-type plants. Under low-P conditions, P concentrations in different organs of the overexpression lines were higher than in the wild type (Figure 10C), but they were slightly lower under sufficient-P conditions (Figure 10D). Then, we calculated total P contents in different organs and found that overexpression of *BnaPT37* significantly reduced P accumulation in seeds under both sufficient and deficient conditions (Figure 10E,F). These results indicate that *BnaPT37* is involved in the transportation of P to seeds in *B. napus*.

## 3. Discussion

The Pi transporter of the PHT1 family has been reported to contain 12 transmembrane domains in many plant species, including a hydrophilic ring between the sixth and seventh transmembrane domains [17,18,40]. The *BnaPT37* gene identified here was found to be homologous with *AtPT4* through phylogenetic tree analysis and sequence comparison, and the amino acid similarity reached more than 98% (Figure 1). Importantly, *AtPT4* has a typical molecular structural feature (GGTYPLSATIMSE) of the PHT1 family [18,44]. The homologous gene *BnaPT37* in *B. napus* was similar, indicating that it may also play an important role in regulating Pi transport (Figure 1). The examination of the three-dimensional protein structure revealed that BnaPT37’s structure was similar to that of *Arabidopsis* AtPT4 (Figure S1), which was consistent with the structural characteristics of PHT1 family genes [17]. Research shows that the PHT1 family encodes a Pi transporter located on the plasma membrane [32,40,45]. The same result was observed here (Figure 3A), indicating that BnaPT37 is a plasma membrane-localized Pi transporter.

PHT1 family members may express in different tissues with varied intensity in response to P deficiency [19,20,21,22,23]. The *PHT1;4* gene in rapeseed was expressed in early cotyledons, and its overexpression significantly reduced the accumulation of P in *B. napus* cotyledons [41]. *AtPHT1;5* is highly expressed in the P-deficient shoot, and the mutation of *AtPHT1;5* affects P allocation to the shoot, indicating that *AtPHT1;5* is involved in P homeostasis in *Arabidopsis* [26]. The abundance of *BnaPT37* was induced by P deficiency in all tissues at the rapeseed seedling stage, but the highest expression levels were observed in leaves, followed by roots (Figure 2). The tissue-specific localization analysis showed that the protein level of BnaPT37 was enhanced in the root, cotyledon, and leaf under P deficiency (Figure 3B). *Arabidopsis AtPHT1;4* is induced by P deficiency in *Arabidopsis* root and shoot, but the expression level in the root is higher than in the shoot. The mutation of *AtPHT1;4* significantly reduces the Pi absorption rate, indicating that it mainly participates in Pi uptake [14]. However, little is known about the function of *AtPT4* in *Arabidopsis*. Here, we speculated based on the expression pattern of the *AtPT4* homologous gene, *BnaPT37*, that it may be involved in Pi translocation and distribution but not in Pi uptake (Figure 2 and Figure 3).

PHT1 family genes mainly transport Pi from soil to root cells through Pi/H^+^ co-transport [12,13,19]. The high-affinity PHT1 Pi transporters can absorb Pi under extremely-low-external-Pi concentration conditions, and their Km values are generally in the micromole range [5,21,28]. PHO84 is the first high-affinity P transporter identified in yeast [46]. The yeast mutant strain with its functional defect is often used to study the transport activity of Pi transporters. We found that *BnaPT37* can restore the growth defects of yeast mutant stain to a certain extent, even when 0.1 mM of Pi is supplied externally (Figure 4). Moreover, we introduced *BnaPT37* to the *Arabidopsis* double-mutant *atpt1/2* and found that the concentrations of inorganic P in the complementary lines were significantly increased, and the complementary lines grew better compared to the double-mutant and the wild type (Figure S2), indicating that *BnaPT37* can transport Pi [29,36,40].

PHT1 family members are mainly up-regulated in the root under P-deficient conditions, but some of them are also expressed in leaf and other tissues, such as *OsPHT1;4*, *BnPHT1;4*, and *GmPHT1;5*, which results in functional differentiation [32,41,47]. *BnaPT37* has the highest homology with *Arabidopsis AtPT4* (Figure 1), and it is induced by P deficiency in both root and shoot, especially in the leaf (Figure 2). It is suggested that *BnaPT37* may not be involved in the Pi uptake, but functions in the Pi translocation or distribution. Previous research showed that the P concentration in the old leaves of the *ospht1;7* mutant was significantly higher than in the wild type at both low and normal P levels. The P allocation to the new leaves of the mutant was significantly reduced compared to the wild type, which affected the development of anthers and ultimately led to a decline in yield [31]. In addition, *Arabidopsis AtPHT1;5* overexpression lines senesced earlier than in the wild type, with chlorosis in old leaves, a significant decrease in P concentration in rosette leaves, and an increase in inflorescence stems and siliques, indicating that *AtPHT1;5* promoted the redistribution of Pi from source to sink [26]. Here, the overexpression of *BnaPT37* led to the accumulation of Pi in plants under high P supplies as well as high inorganic P concentrations (Figure 5). It is noteworthy that under both P supplies, *BnaPT37* overexpression plants showed Pi toxic symptoms (Figure 5A,E). Similar results were observed by overexpressing *OsPHT1;3* and *OsPHT1;8* [28,29]. Moreover, the overexpression of *BnaPT37* significantly reduced P distribution to new leaves compared to that of the wild type (Figure 7). This is different from *ZmPHT1;7* and *OsPHT1;8*. Overexpression of *ZmPHT1;7* led to more P accumulation in new leaves, while overexpression of *OsPHT1;8* significantly increased the inorganic P concentration in new leaves at low P levels [29,36]. Here, we found that overexpression of *BnaPT37* significantly increased the inorganic P concentration in the xylem sap as well as the ratio of root to shoot translocation (Figure 6). Similar results were observed by overexpressing *OsPHT1;4* in rice [32]. In addition, overexpression of *BnaPT37* facilitated the movement of P from a fully expanded leaf to a new leaf and root (Figure 8). This is quite different from the *Arabidopsis* homologous gene previously reported. Furthermore, overexpression of *BnaPT37* decreased flowering time, plant height, and P accumulation in seeds under both P treatments (Figure 9 and Figure 10). However, seed yield was not affected under normal P supply (Figure 10).

Hydroponic culture experiment showed that overexpression of *BnaPT37* significantly increased the transport of Pi from root to shoot, but more Pi was accumulated in cotyledon and old leaves, and the distribution of P to new leaves was significantly reduced (Figure 6 and Figure 7). This is different from *Arabidopsis AtPHT1;5* overexpression lines, which showed a significant decrease in P concentration in rosette leaves, and P in siliques increased significantly [26]. Here, overexpression of *BnaPT37* led to a decrease in yield and P accumulation in seeds under low P conditions, and significantly reduced P accumulation in seeds under normal-P treatment (Figure 10). We speculated that the overexpression of *BnaPT37* in the rapeseed ripening stage might retain more P in the fallen leaves, leading to a decrease in Pi transport to the seeds. Taken together, our data imply that BnaPT37 is a plasma membrane-localized Pi transporter that might be involved in Pi translocation from root to shoot and Pi distribution from source organs to sink organs in *B. napus*.

## 4. Materials and Methods

### 4.1. Plant Materials and Growth Conditions

The *B. napus* cultivar “Westar 10” and the gene overexpression lines were used in this study. The plants were grown in hydroponic conditions in an illuminated culture room. The light intensity was 300–320 μmol m^−2^ s^−1^, the light period was 16 h light/8 h dark, and the temperature was 22 °C. Seeds were surface-sterilized for 12 min using 1% NaClO and washed five times with pure water. Then, the surface-sterilized seeds were immersed for 24 h in deionized water prior to germination on moistened gauze. After 5 d of growth in a solution containing 0.5 mM CaCl_2_, the uniformly sized seedlings were transferred to Hoagland’s nutrient solution [48]. Two P levels including high P (1 mM) and low P (10 μM) were set. The nutrient solution was refreshed every 3 d. The *Arabidopsis* seedlings were grown on a 1/2 MS solid medium [49] and treated with 625 μM Pi and 15 μM Pi conditions, respectively. The *Arabidopsis* seedlings and transgenic materials of *B. napus* were cultured for about 15 days and 19 days, respectively. Then, at least 3 biological replicates were sampled.

For the field trial, the *B. napus* cultivar “Zhongshuang 11” was grown in the field under normal (CK, 90 kg ha^−1^ P_2_O_5_)- and low-P (LP, 15 kg ha^−1^ P_2_O_5_) conditions. Except for P fertilizer, the application rates of other fertilizers were as follows: nitrogen (N) 180 kg N ha^−1^, K_2_O 120 kg ha^−1^ and borax 15 kg ha^−1^. Before sowing, K (potassium chloride) and P (ordinary superphosphate) were applied. N as urea was divided into 120 kg before planting, 30 kg during the seedling stage, and 30 kg at the bolting stage. Different tissues after sampling were used for RNA isolation. Three biological replicates with two plants each were sampled at harvest. For the pot culture, two P treatments were normal (CK, 150 mg kg^−1^ P_2_O_5_) and low P (LP, 20 mg kg^−1^ P_2_O_5_). Both the *BnaPT37* overexpression lines and the wild-type plants were grown in a pot containing 7 kg of soil. The basic properties of the soil were as follows: pH 6.36 (soil:water ratio of 1:2.5), organic matter 5.92 g kg^−1^, alkaline hydrolysis N 9.46 mg kg^−1^, available P 5.98 mg kg^−1^, available potassium 113.38 mg kg^−1^. Four replicates for each treatment were applied. Each pot was soaked in 2000 mL of water containing 3.01 g of KNO_3_, 1.75 g of MgSO_4_·7H_2_O, 4.64 g of (NH_4_)_2_SO_4_ and 7 mL of the Arnon storage solution (1000×), 7 mL of FeSO_4_·EDTA and the Hogland storage solution (200×). P concentration was measured at rapeseed flowering and ripening stages, and agronomic traits and seed yield were analyzed at the ripening stage.

### 4.2. Gene Structure and Sequence Analysis

According to the gene ID of *BnaPT37* (*BnaC04g46050D*) from the “Darmor-*bzh*” genome database (http://www.genoscope.cns.fr/brassicanapus/, accessed on 15 May 2017) [50], the reference genome sequences, CDS, and amino acid sequences were obtained. The amino acid sequence of the *B. napus BnaPT37* gene was compared to that of the homologous gene in *A. thaliana* on the Clustal Omega website (https://www.ebi.ac.uk/Tools/msa/clustalo/, accessed on 20 May 2017). Transmembrane domains and conservative domains were predicted using HMMTOP (http://www.enzim.hu/hmmtop/html/submit.html, accessed on 15 July 2022). The transmembrane domain model was displayed using TOPO2 (http://www.sacs.ucsf.edu/TOPO2/, accessed on 16 July 2022) software. The Expasy website (https://prosite.expasy.org/prosite.html, accessed on 16 July 2022) was used to predict the significant domains. The significant domains were predicted by Expasy (https://prosite.expasy.org/prosite.html, accessed on 16 July 2022). MEGA version 7.0 software was used for phylogenetic relationship analysis with 1000 bootstrap replicates. The CDS and amino acid sequences of the *BnaPT37* gene and the *Arabidopsis* homologous gene were compared by DNAMAN version 5.2.9 software. The SWISS-MODEL online website (https://swissmodel.expasy.org/interactive, accessed on 23 September 2022) was used to predict the three-dimensional structure of proteins and then displayed through ChimeraX-1.1 software.

### 4.3. RNA Extraction and Real-Time Quantitative PCR Analysis

Total RNA extraction of plant samples was performed using the Eastep^®^Super Total RNA Extraction Kit (Qiagen, Promega, Shanghai, China). First-strand cDNA was synthesized using the ReverTra Ace qPCR RT Master Mix with the gDNA Remover Kit (Toyobo, Osaka, Japan). Real-time RT-PCR was performed using the ABI7300 Real-time Detection System (Applied Biosystems, Foster City, CA, USA) with the SYBR Premix Ex TaqTM II (Toyobo, Shanghai, China). The *AtUBC9* and *BnaEF1-a* genes were used as reference genes. The relative expression levels of the target genes were calculated by the 2^−∆∆CT^ method [51]. All the primers used in this study are listed in Table S1.

### 4.4. Vector Construction and Transformation in Arabidopsis and B. napus

For the overexpression of *BnaPT37* in *Arabidopsis* and *B. napus*, the 1605 bp CDS was cloned into the PBI121s vector driven by the 35S promoter. The purified PCR product was digested with XbaI and SmaI and cloned into the PBI121s vector. For the tissue localization vector of *BnaPT37*, a 2000 bp DNA fragment upstream of the translation start codon was amplified from the genomic DNA. The purified PCR product was digested with XmaI and cloned into the DX2181b vector. All constructs were transformed into *Agrobacterium agrobacterium* strain GV3101 by the electroporation method and then transformed into the *Arabidopsis atpt1/2* double mutant [52] and the *B. napus* cultivar “Westar 10” [53]. The *Arabidopsis* seeds were selected on a 1/2 MS medium containing 50 mg of L^−1^ kanamycin. And the *B. napus* transgenic plants were identified by PCR amplification of target fragments. The homozygous lines were used for phenotype analysis. The primers used in this study are listed in Table S1.

### 4.5. Subcellular Localization Analysis

For transient expression analysis in *Arabidopsis* protoplasts, the open reading frame sequence of *BnaPT37* was fused in frame into the position between the 35S promoter and the green fluorescent protein (GFP) sequence in the PM999 plasmid. Because the PM999 vector has a short fragment, the PM999 vector was linearized by PCR amplification with the PM999-GFP-F/R primer, and the target fragment was connected to the vector through In-Fusion cloning. The *Arabidopsis* mesophyll protoplasts were extracted and the *BnaPT37-GFP* vector was transformed into the protoplasts using a PEG-mediated method. The transformation of *Arabidopsis* mesophyll protoplasts was performed using the method described previously [54]. After incubation at room temperature in the dark for 12–16 h, the GFP signal was observed using a laser confocal microscope (TCS SP8; Leica, Wetzlar, Germany). The primers used in this study are listed in Table S1.

### 4.6. Tissue Localization Analysis

In order to clarify the tissue localization of the *BnaPT37* gene, we amplified the 2 kb promoter region of the *BnaPT37* gene and cloned it into the DX2181b vector to construct the *pBnaPT37:GUS* vector. The *pBnaPT37:GUS* transgenic *B. napus* plants, together with the wild-type plants, were cultivated in 1 mM P conditions for 7 days, and then transferred to 1 mM or 0 mM P conditions for additional 7 days. The GUS activity was examined according to the methods described previously [55].

### 4.7. Pi Transport Activity Assay in Yeast

To generate vectors for the yeast complementation test, the coding sequences of *B. napus BnaPT37* and *Arabidopsis* PHO84 were amplified from “Westar 10” and Col-0, respectively, and cloned into the PRS426-ADH1 vector linearized with BamHI and NotI using the In-Fusion HD Cloning kit (PT5162-1; Takara Bio, Kusatsu, Japan). The above constructs and empty vector were transformed independently into the yeast mutant strain EY917, which is defective in the Pi uptake [42]. The vector plasmid was transformed into yeast by the PEG/lithium acetate (LiAc) method. The transformants were screened in the growth medium lacking uracil and tryptophan (−Ura−Trp). The functional complementation assay of BnaPT37 in the yeast mutant strain was performed according to the methods described previously [28]. The OD_600_ values were measured at 15 h, 20 h, 30 h, 35 h, and 40 h, and were used to draw the growth curve. The primers used in this study are listed in Table S1.

### 4.8. Measurement of Inorganic P and Total P Concentrations in Plants

The shoot, root, and other tissues of the wild-type and transgenic seedlings were sampled separately. The Pi concentrations were measured using the malachite green methods described previously [56]. About 25–50 mg of fresh tissue samples was mixed with 25 µL of 5 M H_2_SO_4_ and 1.5 mL of distilled water. After centrifugation at 12,000× *g* at 4 °C, the supernatant was collected and diluted to an appropriate concentration. The diluted supernatant was mixed with a malachite green reaction solution (19.4 mM H_3_BO_3_, 27.64 mM (NH_4_)_6_MO_7_O_24_·4H_2_O, 2.38 M H_2_SO_4_, 627.5 μM malachite green solid reagent, and 0.1% polyvinyl alcohol) at a 3:1 ratio. After the reaction for 30 min, 200 µL of the reaction mixture were taken to measure the absorption values at 650 nm using ELIASA (Spark; TECAN, Männedorf, Switzerland). For the determination of Pi concentrations in samples, a standard curve was developed using varying concentrations of KH_2_PO_4_.

The total P concentrations were measured using the H_2_SO_4_-H_2_O_2_ methods described previously [57]. About 50 mg of dried plant samples were predigested overnight in glass tubes with 2 mL of H_2_SO_4_. Then, the tubes were heated to 120 °C with four to five drops of 30% H_2_O_2_ every 30 min until the solution became colorless. The total P content was measured by molybdenum blue colorimetry at 700 nm using ELISA (Spark; TECAN) after the reaction at 30 °C for 30 min. For the determination of total P content in samples, a standard curve was developed using different concentrations of KH_2_PO_4_.

### 4.9. Xylem Sap Collection

Five-day-old hydroponically grown *B. napus* seedlings were exposed to 1 mM Pi and 10 µM Pi for 14 days; then, xylem sap samples were collected. Briefly, stems were cut with a razor blade 2–3 cm above the basal stems to collect xylem sap. The xylem sap from the first 10 min was discarded to avoid contamination. About 200–300 µL were collected. Each replicate consisted of 8–9 plants, and 5 replicates collected. After a certain dilution of the collected xylem sap, the diluent was mixed with malachite green reaction solution at a 3:1 ratio. After the reaction for 30 min, 200 µL of the reaction mixture was taken to measure the absorption values at 650 nm using ELIASA (Spark; TECAN).

### 4.10. Statistical Analysis of Data

Statistics were performed by Duncan’s test or Student’s *t* test. Data significantly different from those in the corresponding controls are indicated as * *p* < 0.05, ** *p* < 0.01, *** *p* < 0.001.

## Supplementary Materials

The following supporting information can be downloaded at: https://www.mdpi.com/article/10.3390/plants12193362/s1, Figure S1: Three-dimensional protein structure analysis of BnaPT37 in *Brassica napus* and AtPT4 in *Arabidopsis*. The SWISS-MODEL online website was used to predict the three-dimensional structure of proteins. ChimeraX-1.1 software was used for the comparison of the three-dimensional structure of proteins; Figure S2: Overexpression of *BnaPT37* restored the growth of the *Arabidopsis atpt1/2* double mutant. (A) RT-PCR analysis of *BnaPT37* transcripts in *OEBnaPT37-atpt1/2* complementary transgenic lines in *Arabidopsis*; (B) The growth performance of the wild type, mutant, and complementary lines; C-F, Fresh weight (C,D) and Pi concentration (E,F) of Col-0, *atpt1/2* mutant, and *OEBnaPT37-atpt1/2* complementary transgenic lines in 625 μM (C,E) and 15 μM P (D,F) media. The 5-day-old seedlings grown on 1/2 MS medium (625 μM P) were transferred to 625 μM P and 15 μM P media for 10 d. Then, the shoot and root were harvested separately for Pi measurement. Bar = 0.5 cm (625 μM P) or 0.3 cm (15 μM P). Error bars indicate the standard deviation (*n* = 4). Data significantly different from those in the corresponding controls are indicated (*p* < 0.05, Duncan’s test); Figure S3: RT-PCR analysis of *BnaPT37* transcripts in *35S:BnaPT37* transgenic lines in *Brassica napus*; Figure S4: Overexpression of *BnaPT37* affects plant photosynthesis in *Brassica napus*. The 5-day-old seedlings were transferred to 10 μM and 1 mM P conditions for 14 d. Then, the SPDAD values (A,G), chlorophyl concentration (B–D, H–J), carotenoid concentration (E,K), and chlorophyll (a + b)/carotenoid ratio (F,L) of Westar10 and *BnaPT37* overexpression lines were investigated under 10 uM (A–F) and 1 mM P (G–L) conditions in *B. napus*. Error bars indicate the standard deviation (*n* = 4). Data significantly different from those in the corresponding controls are indicated as * *p* < 0.05, ** *p* < 0.01, and *** *p* < 0.001 by Student’s *t* test; Figure S5: The total phosphorus (P) content of Westar10 and *BnaPT37* overexpression plants under two contrasting P conditions. The 5-day-old seedlings were transferred to 10 μM (A) and 1 mM (B) P conditions for 14 d. Error bars indicate the standard deviation (*n* = 4). Data significantly different from those in the corresponding controls are indicated as * *p* < 0.05, ** *p* < 0.01, and *** *p* < 0.001 by Student’s *t* test; Figure S6: Phosphorus (P) concentration, content, and distribution of Westar10 and *BnaPT37* overexpression lines at the rapeseed flowering stage. The Westar10 and *BnaPT37* overexpression plants were grown under normal (CK, 150 mg kg^−1^ P_2_O_5_) and low (LP, 20 mg kg^−1^ P_2_O_5_) P conditions until flowering in a pot trail. Then, stem, lower leaves, upper leaves, and flower bud were sampled separately to determine P concentration (A,D), P content (B,E), and P distribution (C,F) under LP (A–C) and CK (D–F) conditions. The lower leaves contain the first leaf to the sixth leaf, and the upper leaves contain the seventh leaf to the youngest leaf. Each treatment contains four biological replicates (*n* = 4). Error bars indicate the standard deviation. Data significantly different from that in the corresponding controls are indicated as * *p* < 0.05, ** *p* < 0.01, and *** *p* < 0.001 by Student’s *t* test; Figure S7: Agronomic performance of Westar10 and *BnaPT37* overexpression plants under two contrasting phosphorus (P) conditions at the rapeseed ripening stage. (A,B). The growth performance of Westar10 and *BnaPT37* overexpression plants under low (A) and normal (B) P supplies. (C) Plant height. (D) Height of first branch. (E) Branch number. (F) Number of seeds per pod. (G) 1000-seed weight. (H) Pod numbers of main stem. (I) Pod numbers of branch. (J) Pod hull weight. (K) Straw weight. The Westar10 and *BnaPT37* overexpression plants were grown under normal (CK, 150 mg kg^−1^ P_2_O_5_) and low (LP, 20 mg kg^−1^ P_2_O_5_) P conditions until the seed ripened in a pot trail. Error bars indicate the standard deviation (*n* = 8). Data significantly different from those in the corresponding controls are indicated as * *p* < 0.05, ** *p* < 0.01, and *** *p* < 0.001 by Student’s *t* test; Table S1: Primers used for quantitative real-time PCR and vector construction in this study.

## References

[1] Hawkesford M., Horst W., Kichey T., Lamber H., Schjoerring J., Møller I.S., White P. (2012). Chapter 6: Functions of Macronutrients. Marschner’s Mineral Nutrition of Higher Plants.

[2] Song Z.P., Fan N.B., Jiao G.Z., Liu M.H., Wang X.Y., Jia H.F. (2019). Overexpression of OsPT8 Increases Auxin Content and Enhances Tolerance to High-Temperature Stress in Nicotiana tabacum. Genes.

[3] Cao M., Liu H., Zhang C., Wang D., Liu X., Chen Q. (2020). Functional Analysis of *StPHT1;7*, a *Solanum tuberosum* L. Phosphate Transporter Gene, in Growth and Drought Tolerance. Plants.

[4] Kong Y., Wang G., Chen X., Li L., Zhang X., Chen S., He Y., Hong G. (2021). OsPHR2 modulates phosphate starvation-induced OsMYC2 signaling and resistance to *Xanthomonas oryzae* pv. *oryzae*. Plant Cell Environ..

[5] Schachtman D.P., Reid R.J., Ayling S.M. (1998). Phosphorus uptake by plants: From soil to cell. Plant Physiol..

[6] Vance C.P., Uhde-Stone C., Allan D.L. (2003). Phosphorus acquisition and use: Critical adaptations by plants for securing a nonrenewable resource. New Phytol..

[7] Conley D.J., Paerl H.W., Howarth R.W., Boesch D.F., Seitzinger S.P., Havens K.E., Lancelot C., Likens G.E. (2009). Controlling Eutrophication: Nitrogen and Phosphorus. Science.

[8] Baker A., Ceasar S.A., Palmer A.J., Paterson J.B., Qi W., Muench S.P. (2015). Replace, reuse, recycle: Improving the sustainable use of phosphorus by plants. J. Exp. Bot..

[9] Paz-Ares J., Puga M.I., Rojas-Triana M., Martinez-Hevia I., Diaz S., Poza-Carrion C., Minambres M., Leyva A. (2022). Plant adaptation to low phosphorus availability: Core signaling, crosstalks, and applied implications. Mol. Plant.

[10] Raghothama K.G., Karthikeyan A.S. (2005). Phosphate acquisition. Plant. Soil.

[11] Liu D. (2021). Root developmental responses to phosphorus nutrition. J. Integr. Plant Biol..

[12] Wang F., Deng M., Xu J., Zhu X., Mao C. (2018). Molecular mechanisms of phosphate transport and signaling in higher plants. Semin. Cell Dev. Biol..

[13] Victor Roch G., Maharajan T., Ceasar S.A., Ignacimuthu S. (2019). The Role of PHT1 Family Transporters in the Acquisition and Redistribution of Phosphorus in Plants. Crit. Rev. Plant Sci..

[14] Shin H., Shin H., Dewbre G.R., Harrison M.J. (2004). Phosphate transport in *Arabidopsis*: Pht1;1 and Pht1;4 play a major role in phosphate acquisition from both low- and high-phosphate environments. Plant J..

[15] Lapis-Gaza H.R., Jost R., Finnegan P.M. (2014). *Arabidopsis* Phosphate Transporter1 genes *PHT1;8* and *PHT1;9* are involved in root-to-shoot translocation of orthophosphate. BMC Plant Biol..

[16] Wang X., Wang Y., Piñeros M.A., Wang Z., Wang W., Li C., Wu Z., Kochian L.V., Wu P. (2014). Phosphate Transporters OsPHT1;9 and OsPHT1;10 are Involved in Phosphate Uptake in Rice. Plant Cell Environ..

[17] Hirai T., Heymann J.A.W., Maloney P.C., Subramaniam S. (2003). Structural Model for 12-Helix Transporters Belonging to the Major Facilitator Superfamily. J. Bacteriol..

[18] Karandashov V., Bucher M. (2005). Symbiotic Phosphate Transport in Arbuscular Mycorrhizas. Trends Plant Sci..

[19] Mudge S.R., Rae A.L., Diatloff E., Smith F.W. (2002). Expression analysis suggests novel roles for members of the Pht1 family of phosphate transporters in *Arabidopsis*. Plant J..

[20] Ai P., Sun S., Zhao J., Fan X., Xin W., Guo Q., Yu L., Shen Q., Wu P., Miller A.J. (2009). Two rice phosphate transporters, OsPht1;2 and OsPht1;6, have different functions and kinetic properties in uptake and translocation. Plant J..

[21] Qin L., Guo Y., Chen L., Liang R., Gu M., Xu G., Zhao J., Walk T., Liao H. (2012). Functional Characterization of 14 Pht1 Family Genes in Yeast and Their Expressions in Response to Nutrient Starvation in Soybean. PLoS ONE.

[22] Chen A., Chen X., Wang H., Liao D., Gu M., Qu H., Sun S., Xu G. (2014). Genome-wide investigation and expression analysis suggest diverse roles and genetic redundancy of Pht1 family genes in response to Pi deficiency in tomato. BMC Plant Biol..

[23] Teng W., Zhao Y.-Y., Zhao X.-Q., He X., Ma W.-Y., Deng Y., Chen X.P., Tong Y.-P. (2017). Genome-wide identification, characterization, and expression analysis of PHT1 phosphate transporters in wheat. Front. Plant Sci..

[24] Ayadi A., David P., Arrighi J.F., Chiarenza S., Thibaud M.C., Nussaume L., Marin E. (2015). Reducing the genetic redundancy of Arabidopsis PHOSPHATE TRANSPORTER1 transporters to study phosphate uptake and signaling. Plant Physiol..

[25] Chien P., Chao Y.T., Chou C., Hsu Y., Chiang S., Tung C., Chiou T. (2022). Phosphate transporter PHT1;1 is a key determinant of phosphorus acquisition in *Arabidopsis* natural accessions. Plant Physiol..

[26] Nagarajan V.K., Jain A., Poling M.D., Lewis A.J., Raghothama K.G., Smith A.P. (2011). Arabidopsis Pht1;5 mobilizes phosphate between source and sink organs and influences the interaction between phosphate homeostasis and ethylene signaling. Plant Physiol..

[27] Sun S., Gu M., Cao Y., Huang X., Zhang X., Ai P., Zhao J., Fan X., Xu G. (2012). A constitutive expressed phosphate transporter, OsPht1;1, modulates phosphate uptake and translocation in phosphate-replete Rice. Plant Physiol..

[28] Chang M.X., Gu M., Xia Y.W., Dai X.L., Dai C.R., Zhang J., Wang S.C., Qu H.Y., Yamaji N., Ma J.F. (2019). OsPHT1;3 mediates uptake, translocation, and remobilization of phosphate under extremely low phosphate regimes. Plant Physiol..

[29] Jia H., Ren H., Gu M., Zhao J., Sun S., Zhang X., Chen J., Wu P., Xu G. (2011). The phosphate transporter gene *OsPht1;8* is involved in phosphate homeostasis in Rice. Plant Physiol..

[30] Jia H., Zhang S., Wang L., Yang Y., Zhang H., Cui H., Shao H., Xu G. (2017). OsPht1;8, a phosphate transporter, is involved in auxin and phosphate starvation response in rice. J. Exp. Bot..

[31] Dai C., Dai X., Qu H., Men Q., Liu J., Yu L., Gu M., Xu G. (2022). The rice phosphate transporter *OsPHT1;7* plays a dual role in phosphorus redistribution and anther development. Plant Physiol..

[32] Zhang F., Sun Y., Pei W., Jain A., Sun R., Cao Y., Wu X., Jiang T., Zhang L., Fan X. (2015). Involvement of OsPht1;4 in Phosphate Acquisition and Mobilization Facilitates Embryo Development in Rice. Plant J..

[33] Paszkowski U., Kroken S., Roux C., Briggs S.P. (2002). Rice phosphate transporters include an evolutionarily divergent gene specifically activated in *arbuscular mycorrhizal* symbiosis. Proc. Natl. Acad. Sci. USA.

[34] Yang S.Y., Gronlund M., Jakobsen I., Grotemeyer M.S., Rentsch D., Miyao A., Hirochika H., Kumar C.S., Sundaresan V., Salamin N. (2012). Nonredundant Regulation of Rice Arbuscular Mycorrhizal Symbiosis by Two Members of the PHOSPHATE TRANSPORTER1 Gene Family. Plant Cell.

[35] Młodzińska E., Zboińska M. (2016). Phosphate uptake and allocation-a closer look at *Arabidopsis thaliana* L. and *Oryza sativa* L. Front. Plant Sci..

[36] Wang F., Cui P.J., Tian Y., Huang Y., Wang H.F., Liu F., Chen Y.F. (2020). Maize *ZmPT7* regulates Pi uptake and redistribution which is modulated by phosphorylation. Plant Biotechnol. J..

[37] Ding G., Zhao Z., Liao Y., Hu Y., Shi L., Long Y., Xu F. (2012). Quantitative trait loci for seed yield and yield-related traits, and their responses to reduced phosphorus supply in *Brassica napus*. Ann. Bot..

[38] Wang W., Ding G.-D., White P.J., Wang X.-H., Jin K.-M., Xu F.-S., Shi L. (2019). Mapping and cloning of quantitative trait loci for phosphorus efficiency in crops: Opportunities and challenges. Plant Soil.

[39] Li Y., Wang X., Zhang H., Wang S.L., Ye X.S., Shi L., Xu F.S., Ding G.D. (2019). Molecular identification of the phosphate transporter family 1 (PHT1) genes and their expression profiles in response to phosphorus deprivation and other abiotic stresses in *Brassica napus*. PLoS ONE.

[40] Ren F., Zhao C.Z., Liu C.S., Huang K.L., Guo Q.Q., Chang L.L., Xiong H., Li X.-B. (2014). A Brassica napus PHT1 phosphate transporter, BnPht1;4, promotes phosphate uptake and affects roots architecture of transgenic Arabidopsis. Plant Mol. Biol..

[41] Huang K.-L., Wang H., Wei Y.-L., Jia H.-X., Zha L., Zheng Y., Ren F., Li X.-B. (2019). The high-affinity transporter BnPHT1; 4 is involved in phosphorus acquisition and mobilization for facilitating seed germination and early seedling growth of *Brassica napus*. BMC Plant Biol..

[42] Wykoff D.D., O’Shea E.K. (2001). Phosphate transport and sensing in *Saccharomyces cerevisiae*. Genetics.

[43] Wang C., Yue W., Ying Y., Wang S., Secco D., Liu Y., Whelan J., Tyerman S.D., Shou H. (2015). Rice SPX-Major Facility Superfamily3, a vacuolar phosphate efflux transporter, is involved in maintaining phosphate homeostasis in Rice1. Plant Physiol..

[44] Nussaume L., Kanno S., Javot H., Marin E., Pochon N., Ayadi A., Nakanishi T.M., Thibaud M.C. (2011). Phosphate Import in Plants: Focus on the PHT1 Transporters. Front. Plant Sci..

[45] Chen L., Qin L., Zhou L., Li X., Chen Z., Sun L., Wang W., Lin Z., Zhao J., Yamaji N. (2019). A nodule-localized phosphate transporter *GmPT7* plays an important role in enhancing symbiotic N_2_ fixation and yield in soybean. New Phytol..

[46] Bunya M., Nishimura M., Harashima S., Oshima Y. (1991). The PHO84 gene of *Saccharomyces-cerevisiae* encodes an inorganic-phosphate transporter. Mol. Cell. Biol..

[47] Qin L., Zhao J., Tian J., Chen L., Sun Z., Guo Y., Lu X., Gu M., Xu G., Liao H. (2012). The High-Affinity Phosphate Transporter *GmPT5* Regulates Phosphate Transport to Nodules and Nodulation in Soybean. Plant Physiol..

[48] Hoagland D.R., Arnon D.I. (1950). The Water Culture Method for Growing Plant without Soil. Circular 347.

[49] Murashige T., Skoog F. (1962). A revised medium for rapid growth and bio assays with tobacco tissue cultures. Physiol. Plant..

[50] Chalhoub B., Denoeud F., Liu S., Parkin I.A., Tang H., Wang X., Chiquet J., Belcram H., Tong C., Samans B. (2014). Plant genetics. Early allopolyploid evolution in the post-Neolithic *Brassica napus* oilseed genome. Science.

[51] Livak K.J., Schmittgen T.D. (2001). Analysis of relative gene expression data using real-time quantitative PCR and the 2*^−ΔΔCT^* method. Methods.

[52] Clough S.J., Bent A.F. (1998). Floral dip: A simplified method for Agrobacterium-mediated transformation of *Arabidopsis thaliana*. Plant J..

[53] De Block M., De Brouwer D., Tenning P. (1989). Transformation of *Brassica napus* and *Brassica oleracea* Using Agrobacterium tumefaciens and the Expression of the bar and neo Genes in the Transgenic Plants. Plant Physiol..

[54] Yoo S.D., Cho Y.H., Sheen J. (2007). Arabidopsis mesophyll protoplasts: A versatile cell system for transient gene expression analysis. Nat. Protoc..

[55] Han B., Wang C., Wu T., Yan J., Jiang A., Liu Y., Luo Y., Cai H., Ding G., Dong X. (2022). Identification of vacuolar phosphate influx transporters in *Brassica napus*. Plant Cell Environ..

[56] Lu L., Qiu W., Gao W., Tyerman S.D., Shou H., Wang C. (2016). OsPAP10c, a novel secreted acid phosphatase in rice, plays an important role in the utilization of external organic phosphorus. Plant Cell Environ..

[57] Wang S., Zhang H., Shi L., Xu F., Ding G. (2020). Genome-wide dissection of the CRF gene family in *Brassica napus* indicates that bnacrf8s specifically regulate root architecture and phosphate homeostasis against phosphate fluctuation in plants. Int. J. Mol. Sci..

