# Supplementary material for: Phosphate Transporter BnaPT37 Regulates Phosphate Homeostasis in Brassica napus by Changing Its Translocation and Distribution In Vivo
Source: Plants (Basel). 2023 Sep 22;12(19):3362. doi: 10.3390/plants12193362 (PMC10574216; doi:10.3390/plants12193362)
Supplement: Supplementary file 1 [file plants-12-03362-s001.zip › BnaPT37-Supplementary Figures.pptx]

## Slide 1
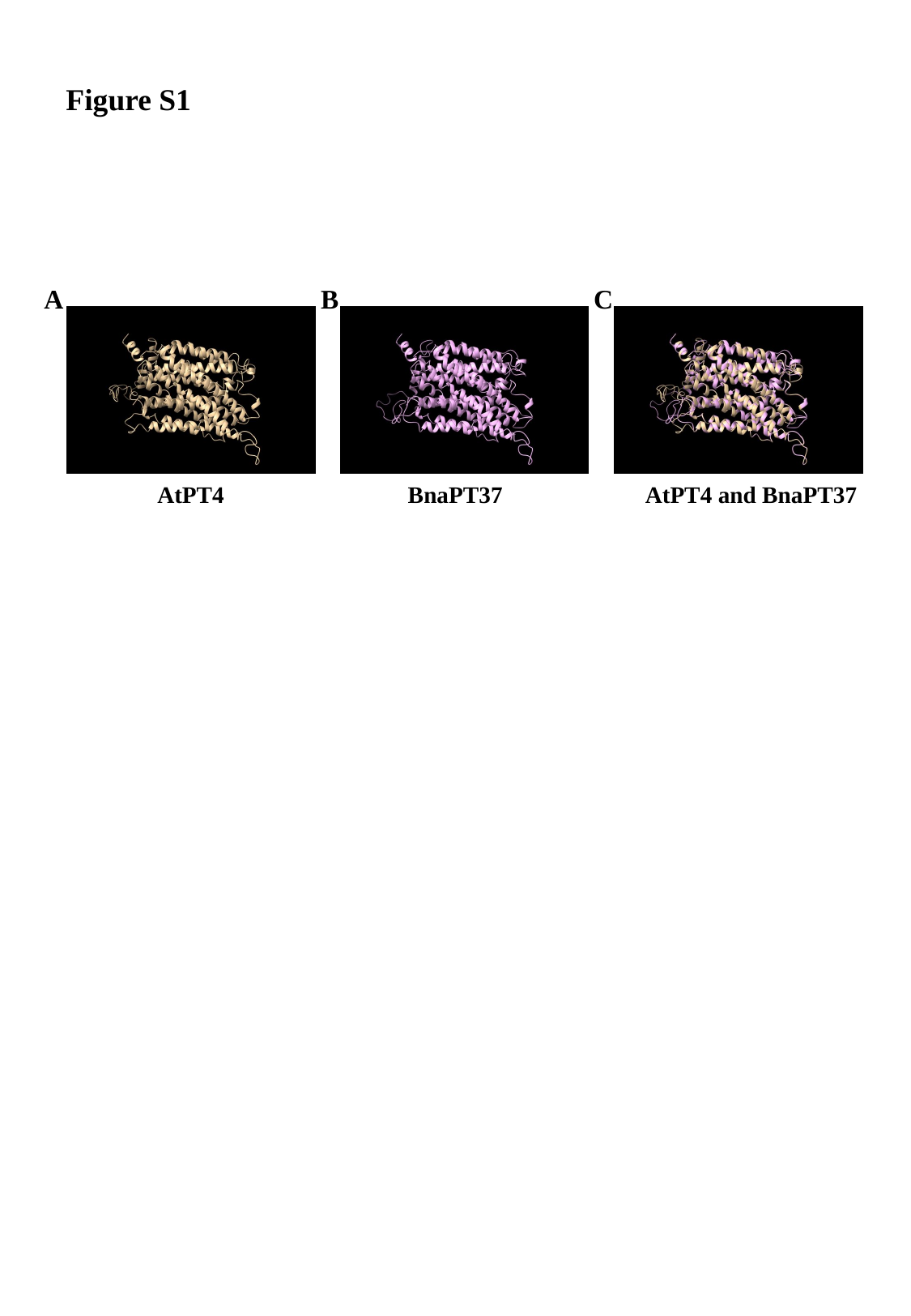

Figure S1
A
B
C
AtPT4
BnaPT37
AtPT4 and BnaPT37

## Slide 2
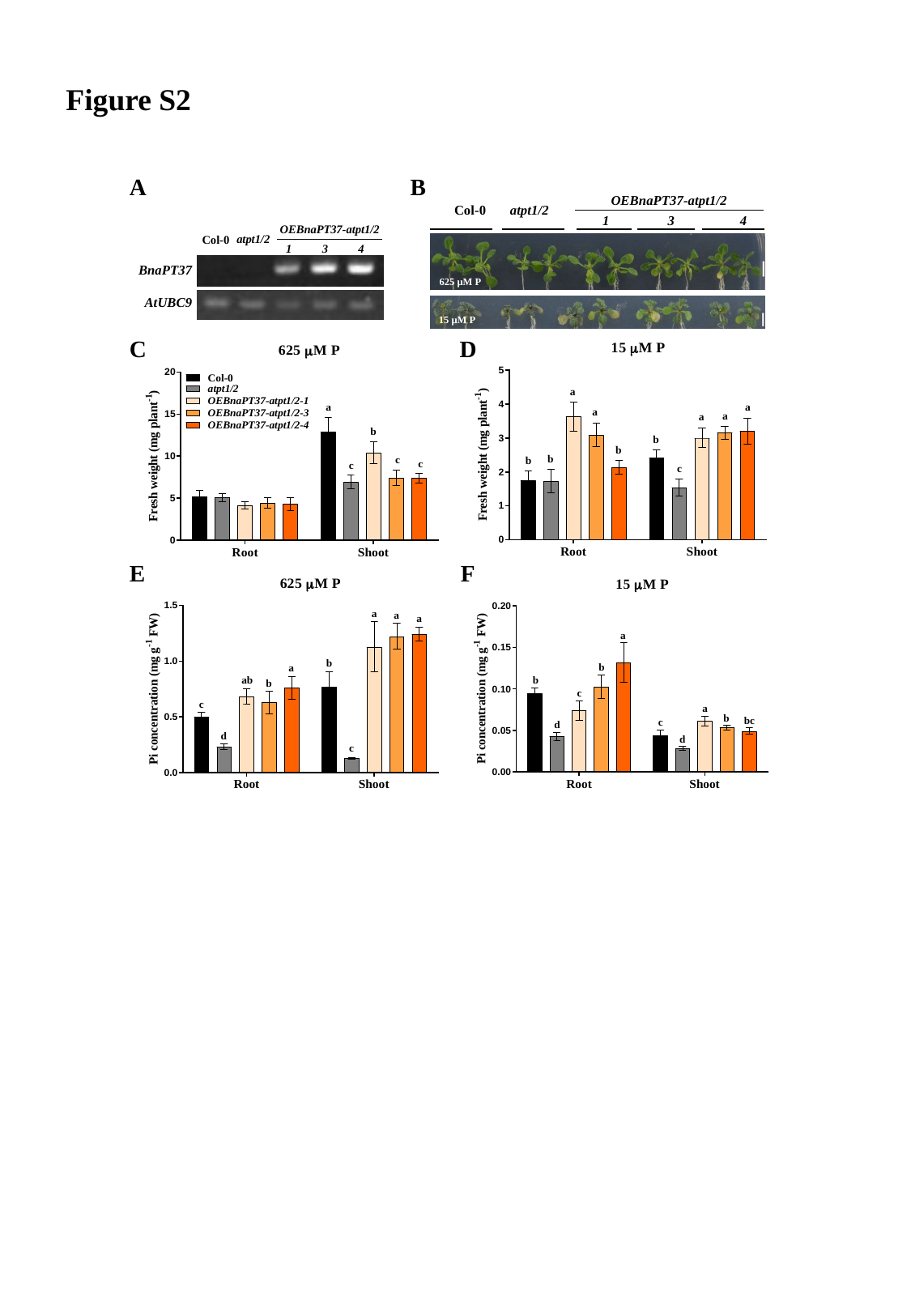

Figure S2
A
B
OEBnaPT37-atpt1/2
Col-0
atpt1/2
 1 3 4
625 μM P
15 μM P
OEBnaPT37-atpt1/2
atpt1/2
Col-0
1 3 4
BnaPT37
AtUBC9
C
D
E
F

## Slide 3
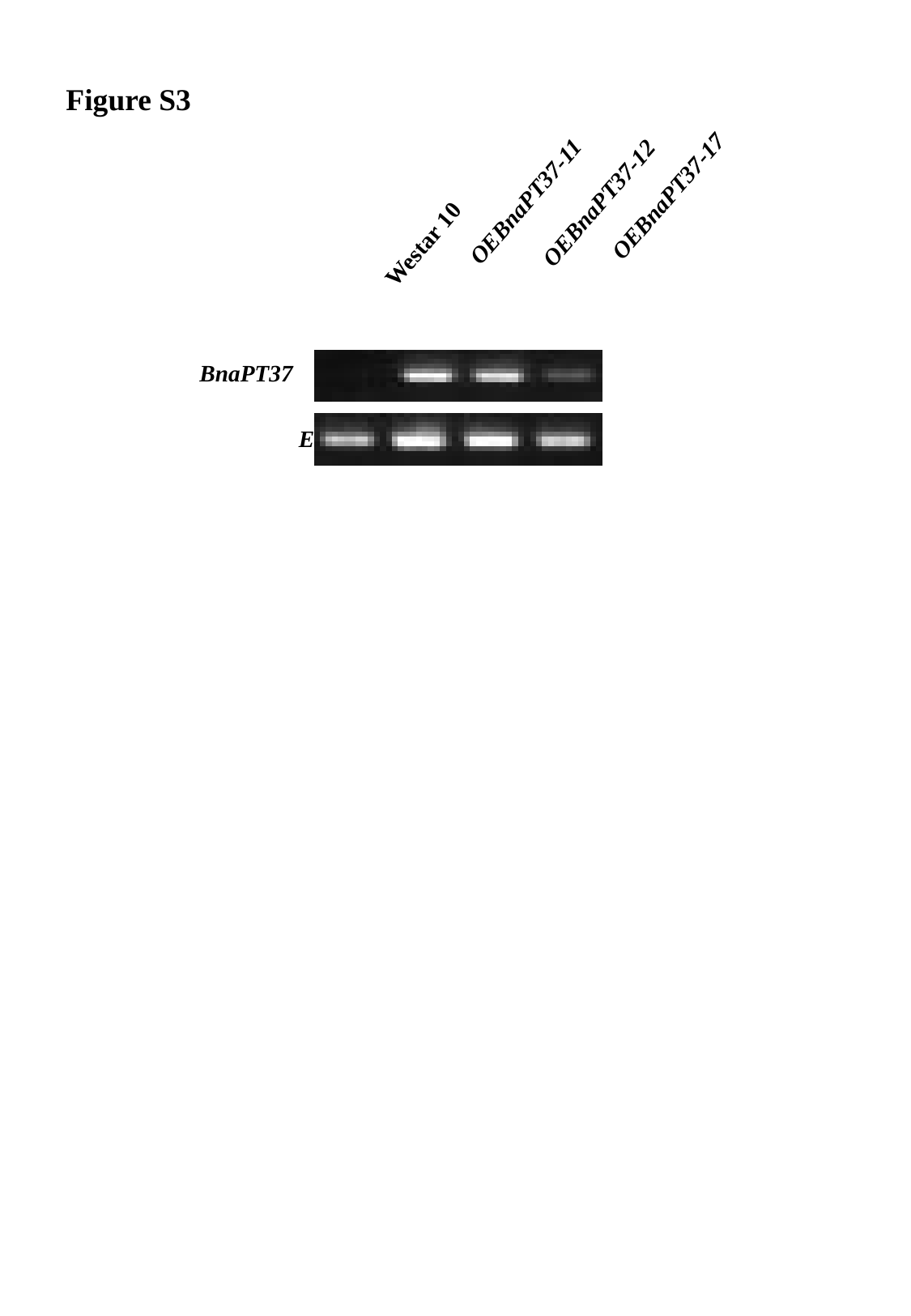

OEBnaPT37-17
OEBnaPT37-11
OEBnaPT37-12
Westar 10
BnaPT37
EF1-a
Figure S3

## Slide 4
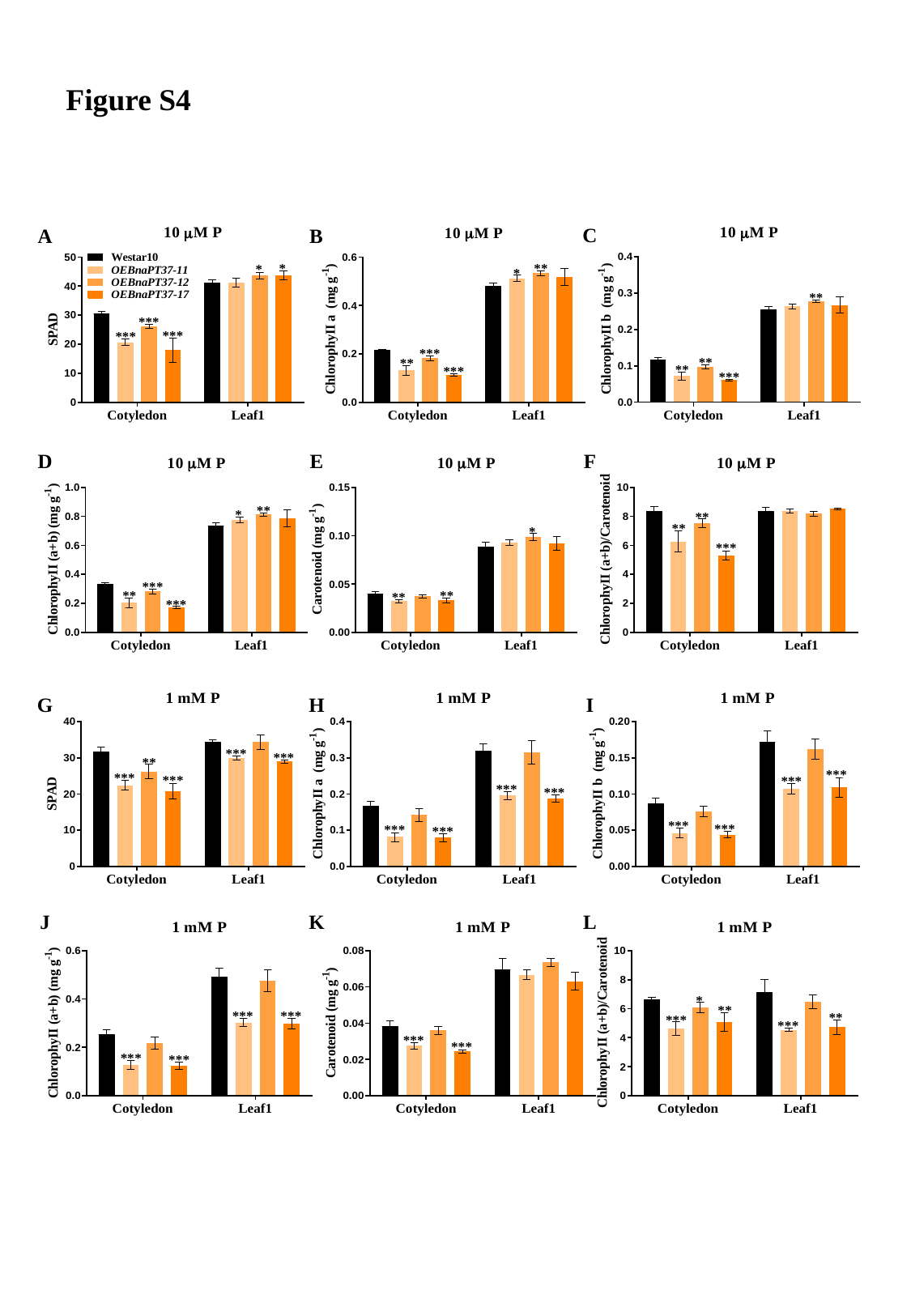

Figure S4
C
A
B
D
E
F
G
H
I
J
K
L

## Slide 5
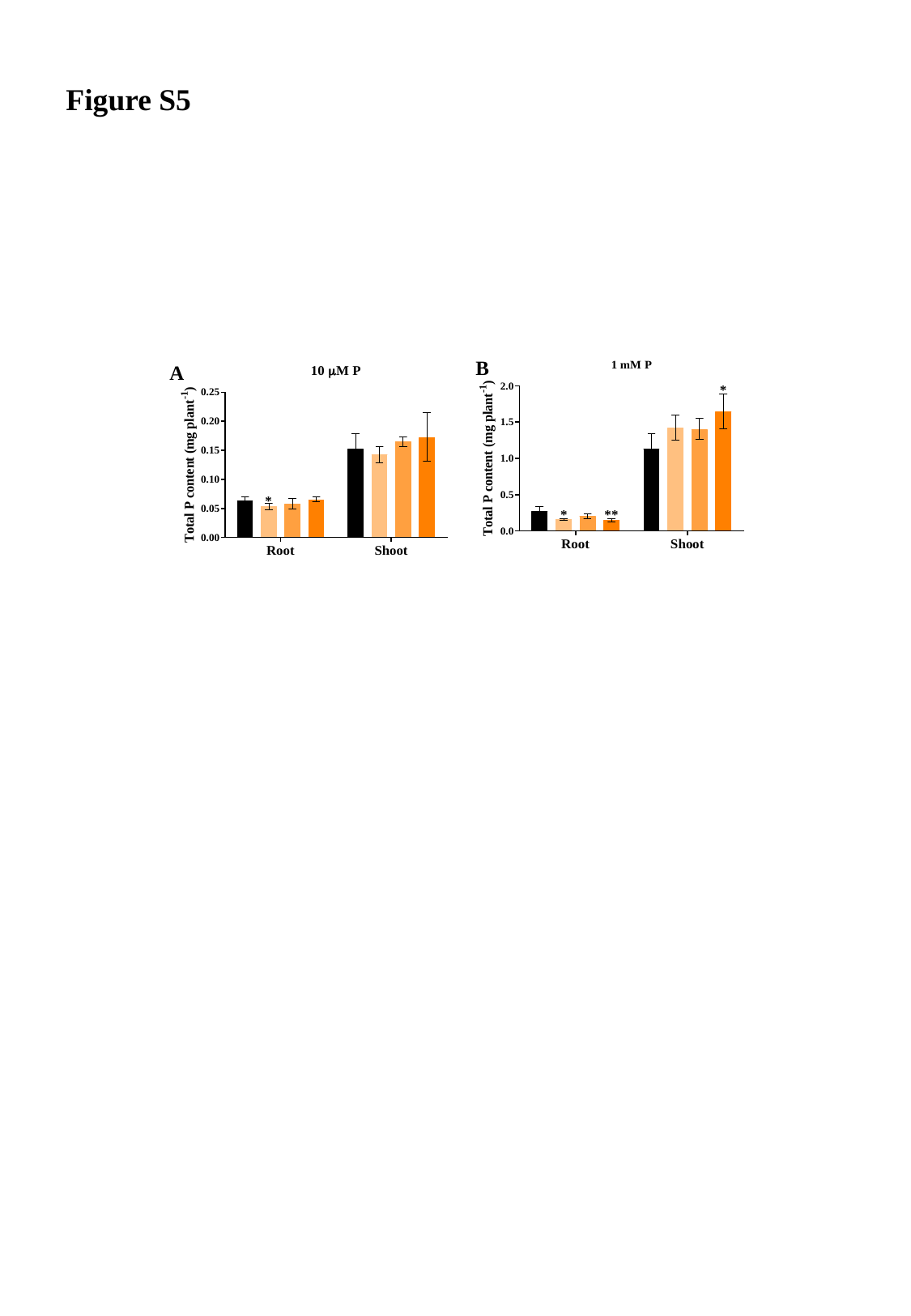

Figure S5
B
A

## Slide 6
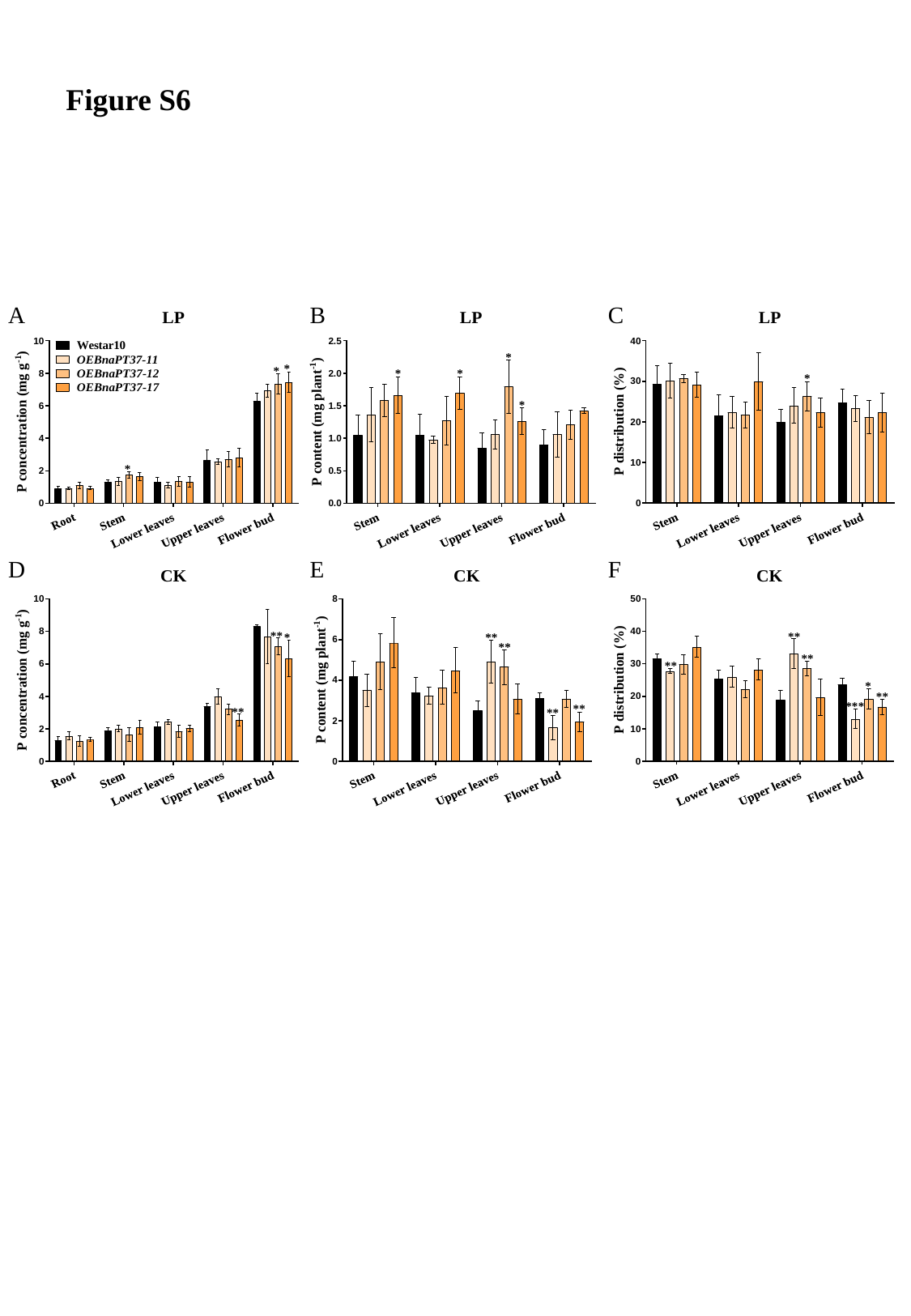

Figure S6
A
B
C
D
E
F

## Slide 7
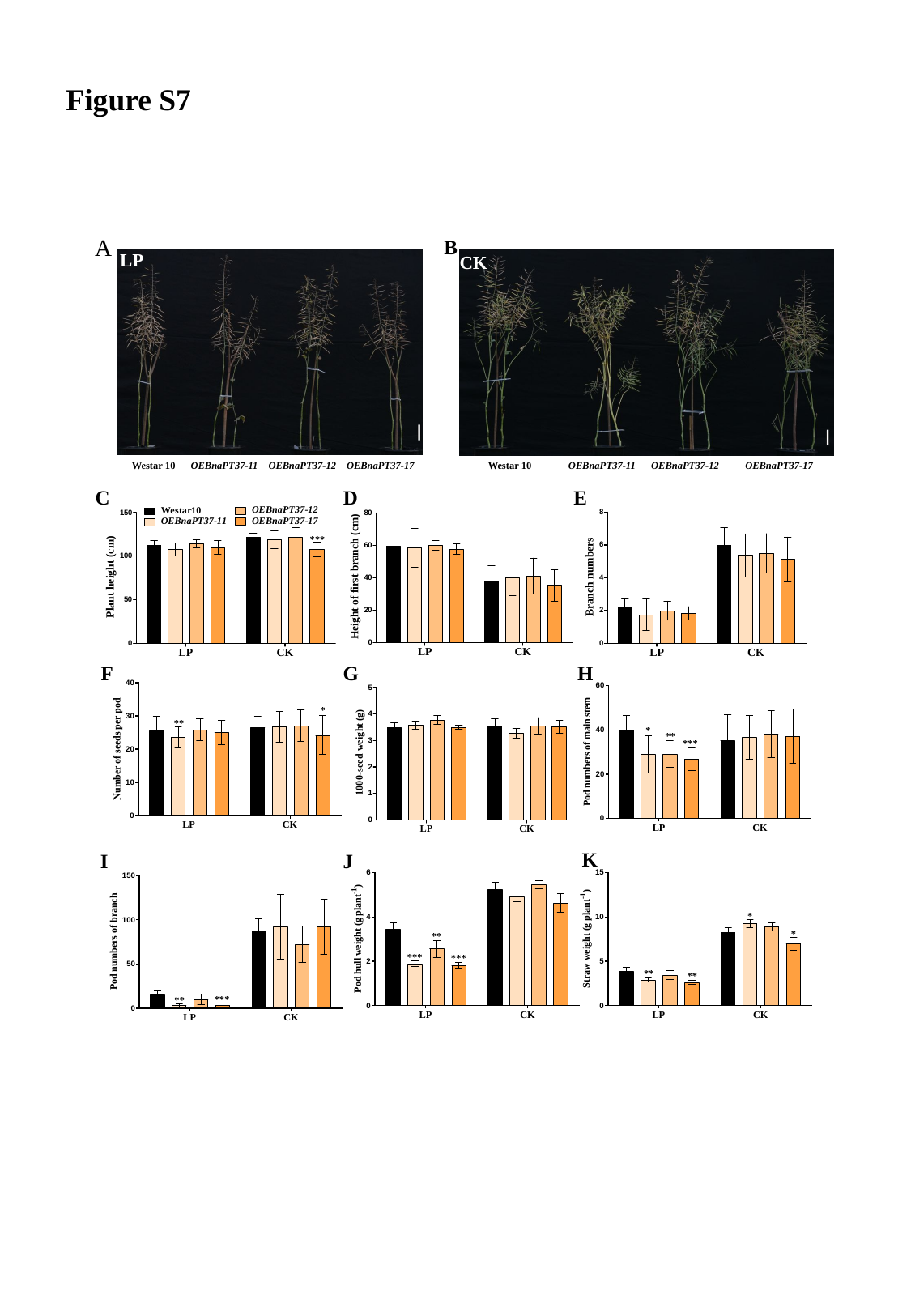

Figure S7
A
B
LP
CK
Westar 10 OEBnaPT37-11 OEBnaPT37-12 OEBnaPT37-17
Westar 10 OEBnaPT37-11 OEBnaPT37-12 OEBnaPT37-17
C
D
E
F
G
H
K
I
J
