# Supplementary material for: Phosphate Transporter BnaPT37 Regulates Phosphate Homeostasis in Brassica napus by Changing Its Translocation and Distribution In Vivo
Source: Plants (Basel). 2023 Sep 22;12(19):3362. doi: 10.3390/plants12193362 (PMC10574216; doi:10.3390/plants12193362)
Supplement: Supplementary file 1 [file plants-12-03362-s001.zip › BnaPT37-Table S1.pdf]

Table S1. Primers used for quantitative real-time PCR and vector construction in this study

Primer name	Primer squence (5' to 3')
BnaPT37-GFP-F	ATCGATTCTAGAGCC ATGGCAAGGGATCAATTACAAGTG
BnaPT37-GFP-R	TTCTCCTTTGCCCAT AACTATTGGGACCGTTCTACTATCGC
PM999-GFP-F	ATGGGCAAAGGAGAAGAACT
PM999-GFP-R	GGCTCTAGAATCGATAGATCT
BnaPT37p-GUS-F	CAGGTCGACGGATCC GGTAAAAAAGAAATGAAGACTCTTG
BnaPT37p-GUS-R	TAAGGGACTGACCAC TATTCTTCACCTCTGTAATTTCATC
KO-yeast-F	CGC GGATCC ACTAGTTA GCGGCCGC TTA
KO-yeast-R	TAA GCGGCCGC TAACTAGT GGATCC GCG
BnaPT37-yeast-F	TCCTGCAGCCCGGGG ATGGCAAGGGATCAATTACAAGTG
BnaPT37-yeast-R	CTCCACCGCGGTGGC CTAAACTATTGGGACCGTTCTACT
BnaPT37OX-F	CCCCCGGGGAATAATGGCAAGGGATCAATTACAAGTGC
BnaPT37OX-R	GCTCTAGATCTAAACTATTGGGACCGTTCTACTATCGCTGTTGC
BnaPT37-qPCR-F	TCGGGAGGAAGAAAGTTTATGG
BnaPT37-qPCR-R	TGATCGTTGCAGACAAAGGGTAG
BnaEF1-*a*-F	GCCTGGTATGGTTGTGACCT
BnaEF1-*a*-R	GAAGTTAGCAGCACCCTTGG
AtUBC9-F	CCAAGGTGCTGCTATCGATCTGT
AtUBC9-R	AGGTCCGAGCAGTGGACTCG
